# Identification of chicken LOC420478 as Bucky ball equivalent and potential germ plasm organizer in birds

**DOI:** 10.1038/s41598-022-21239-8

**Published:** 2022-10-07

**Authors:** Sabine Klein, Roland Dosch, Stefanie Altgilbers, Wilfried A. Kues

**Affiliations:** 1 Department of Biotechnology, Stem Cell Physiology, Institute of Farm Animal Genetics, Friedrich-Loeffler-Institut, Höltystr. 10, Mariensee, 31535 Neustadt, Germany; 2 Institut für Humangenetik, Georg-August-Universität Göttingen, Göttingen, Germany

**Keywords:** Cell biology, Developmental biology, Evolution, Stem cells

## Abstract

Bucky ball was identified as germ plasm organizer in zebrafish and has proven crucial for Balbiani body condensation. A synteny comparison identified an uncharacterized gene locus in the chicken genome as predicted avian counterpart. Here, we present experimental evidence that this gene locus indeed encodes a ‘Bucky ball’ equivalent in matured oocytes and early embryos of chicken. Heterologous expression of Bucky ball fusion proteins both from zebrafish and chicken with a fluorescent reporter revealed unique patterns indicative for liquid–liquid phase separation of intrinsically disordered proteins. Immuno-labeling detected Bucky ball from oocytes to blastoderms with diffuse distribution in matured oocytes, aggregation in first cleavage furrows, and co-localization to the chicken vasa homolog (CVH). Later, Bucky ball translocated to the cytoplasm of first established cells, and showed nuclear translocation during the major zygotic activation together with CVH. Remarkably, during the phase of area pellucida formation, Bucky ball translocated back into the cytoplasm at stage EGK VI, whereas CVH remained within the nuclei. The condensation of Bucky ball and co-localization with CVH in cleavage furrows and nuclei of the centrally located cells strongly suggests chicken Bucky ball as a germ plasm organizer in birds, and indicate a special importance of the major zygotic activation for germline specification.

## Introduction

The germ plasm-based specification of the germline is one of two known principal modes of germ line specification^1–4^ in species as diverse as *Drosophila sp.*, zebrafish (*Danio rerio)*^4–8^, and chicken (*Gallus gallus dom*.)^9^. Germ plasm is composed of maternal RNAs, RNA-binding proteins aggregated with mitochondria and Golgi apparatus in a membrane-less compartment, the Balbiani body, in the cytoplasm of early oocytes, and is exclusively inherited to the germline cells^10^. Recently, Bucky ball, an intrinsically disordered protein (IDP), was described as a germ plasm organizer protein in zebrafish^11–13^. Bucky ball provided functional equivalence to Oskar in *Drosophila*, although there is extremely low sequence homology between Oskar and Bucky ball^8,11–16^. For the separation of the germ plasm from the rest of cytoplasm, the IDP Bucky ball provides essential liquid–liquid phase separation properties^11,17^. In zebrafish mutants lacking a short, 23 amino acid sequence of Bucky ball, localized Vasa activation is not observed and germ cell specification is disrupted^12,18^.

A synteny-based comparison of zebrafish and chicken genomes identified a putative avian counterpart (LOC 420748) of Bucky ball, albeit with no apparent nucleotide homology. The putative chicken Bucky ball gene contained an intact reading frame, whereas some synteny-predicted mammalian Bucky ball sequences do not appear to code for proteins^12^.

In chicken, as in other species with germline inheritance, primordial germ cells (PGCs) specify before gastrulation^2,5,6,19^.The condensation of germ plasm in the cytoplasm is the essential prerequisite for this selective process.

Predisposed PGCs could already be distinguished within the blastoderm in chicken and used for chimera experiments from the laid egg onwards, as reviewed by Costa et al.^20^. The transfer of presumptive PGCs from blastoderm or the primitive streak stage into recipient embryos resulted in germline transfer from the donor^21^. Thus, predefined germline cells must have established before oviposition during the intrauterine stages of development in chickens. Raucci et al.^22^ reported stable expression of the germline marker *chicken vasa homolog (CVH)*, the stem cell marker stage-specific embryonic antigen (SSEA1), and embryonic primordial germ cell marker (EMA1) from blastodermal to gonadal germline stem cells. Lee et al. identified a collection of PGC-specific miRNAs differentially expressed to blastodermal and embryonic stem cells^23^. Additionally, *deleted in azoospermia-like (DAZL)* was identified as a marker of primordial germ cells^24^.

Here we investigated, whether the chicken sequence LOC 420748 actually represents features of a germ plasm organizer. First, we developed an assay to identify proteins with liquid phase separation properties. Therefore, the putative chicken Bucky ball gene was fused in-frame with a fluorescent reporter and was used for ectopic expression analysis. Both the zebrafish and the chicken reporter constructs revealed unique condensation pattern resembling liquid–liquid phase separation. Expression analysis of chicken embryos by reverse transcription—PCR revealed that the Bucky ball mRNA is only present until oviposition. Immuno-histochemical studies using two cross-reactive antibodies raised against zebrafish-Bucky ball^13^, identified the spatio-temporal expression pattern of Bucky ball in chicken intrauterine embryos, strongly supporting the notion that Bucky ball is indeed a germ plasm organizer in this species.

## Results

### Identification of a putative Bucky ball homolog in the chicken genome with the potential of germ plasm organizer function

**Figure 1:**
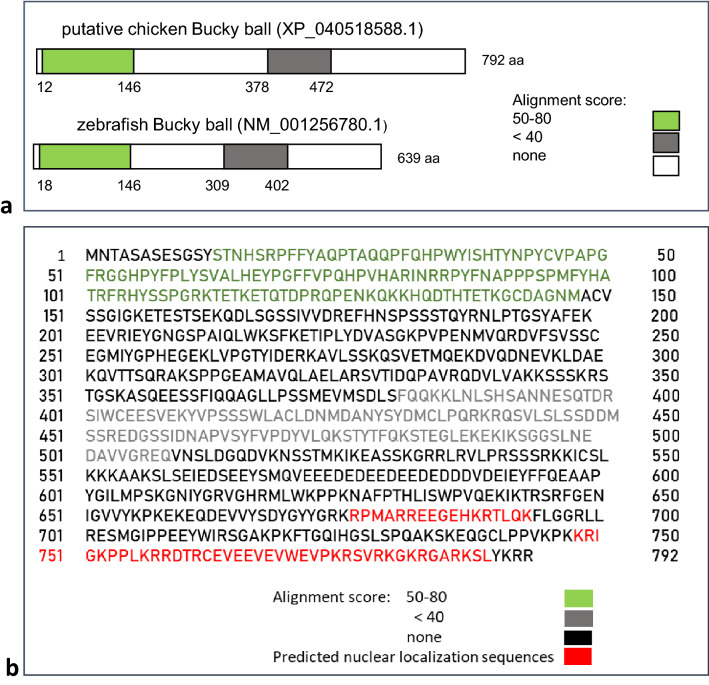
Regions of partial amino acid homology for chicken and zebrafish Bucky ball. (**a**) Homology regions between the predicted chicken (upper panel) and zebrafish Bucky ball amino acid sequences (lower panel). (**b**) Amino acid sequence of LOC 420748 with marked strings of homology to the zebrafish Bucky ball sequence and with highlighted positions of the predicted nuclear localization sequences. The nuclear localization sequences were predicted by cNLS mapper (https://nls-mapper.iab.keio.ac.jp/cgi-bin/NLS_Mapper_form.cg).

Previously, the Bucky ball gene was identified as a germ plasm organizer in zebrafish^11,14,15^. A synteny-based comparison of zebrafish and several vertebrate genomes identified a putative chicken Bucky ball sequence (*Gallus gallus* uncharacterized LOC420748, Supplementary Table S1), but a nucleotide BLAST of chicken Bucky ball (2379 nts. Fig. S1) and zebrafish Bucky ball (1993 nts) cDNAs did not yield any homology (not shown). However, a protein BLAST (blastp) identified two homologous protein domains of 134 amino acids (domain 1) and 96 amino acids (domain 2), (Fig. 1; Supplementary Fig. S2). Interestingly, both Bucky ball proteins contained consensus sequences for nuclear localization signals (Fig. 1, Supplementary Fig. S2)^25^.

To assess, whether the chicken Bucky ball sequence indeed represents a germ plasm organizer, we developed an assay to identify proteins with liquid phase separation properties. Therefore the chicken Bucky ball gene was fused in-frame with a fluorescent reporter gene (Venus) into a eukaryotic expression plasmid (Supplementary Fig. S3), and was used for ectopic expression in cultured chicken cells. In parallel, transfections were done with a zebrafish reporter fusion construct^12^ . Both, the zebrafish and the chicken reporter constructs revealed unique condensation patterns of the fusion proteins reminiscent of liquid–liquid phase separation for all three cell types (Supplementary Fig. S4A–F) in contrast to control transfections (Supplementary Fig. S4G, H).

**Figure 2:**
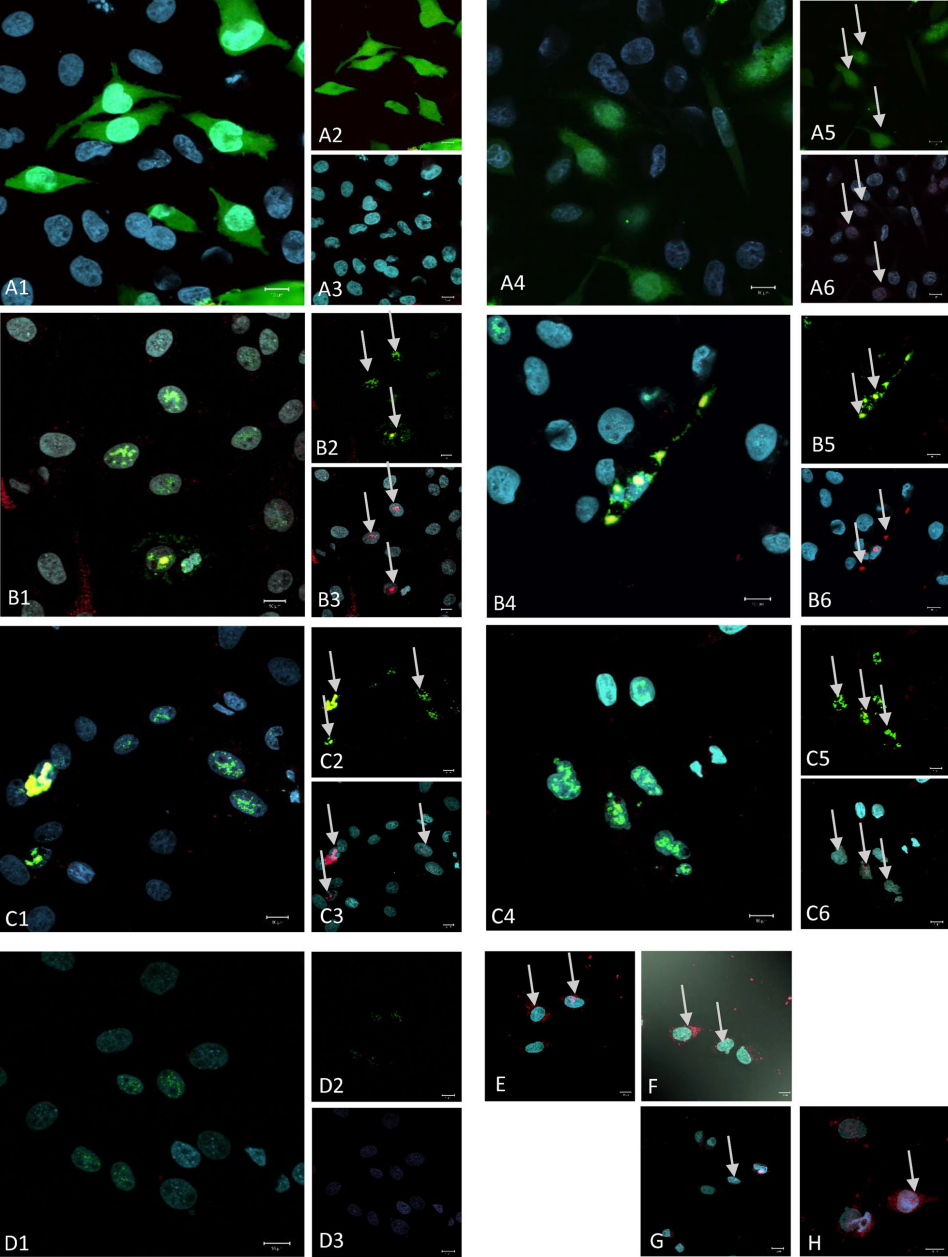
High resolution confocal imaging of single channels for the reporter fluorescence (green fluorescence proteins, green label), for immunocytochemical Bucky ball reactivity in DF1 cells after transfection with different fusion plasmids (red label) and nuclear label using SIR (cyan label): (**A**) two examples for the GFP only plasmid; (**B**) two examples for the chicken Bucky ball—venus fusion plasmid; (**C**) two examples for the zebrafish Bucky ball- GFP fusion plasmid; D negative control sample of chicken Bucky ball transfected cells incubated without primary Bucky ball antibody; Left larger frames represents the overlay of all three channels (1,4), right upper frames represents the overlay of the GFP reporter and Bucky ball channel (2,5), right lower frames (3,6) represent the overlay of the nuclear marker and Bucky ball label). (**E,F**) Examples of DF1 cells transfected with chicken Bucky ball plasmid without Venus reporter; (**G,H**) examples of DF1 cells transfected with zebrafish Bucky ball without GFP reporter, overlay of all three channels each; all images were taken with a 63× oil immersion 1.6 NA objective.

Confocal imaging and counter-staining with the anti-Buc antibody revealed co-localization of the direct GFP fluorescence and Bucky ball immuno-staining in the condensation spots, respectively (Fig. 2).

In parallel, the DF1 cells were transfected with the Bucky ball expression plasmids without reporter. Again, the anti-Buc staining resulted in comparable condensation signals indicated by arrows in Fig. 2E–H). To confirm the specificity of the anti-Buc immune-reactivity, several controls were included. DF1 cells transfected with EGFP/Venus reporter only (Fig. 2A) did not show any reactivity with the anti Buc antibody. Also, when cells transfected with Bucky ball construct were processed for immunostaining but omitting the primary antibody, no background in the red channel was found (Fig. 2D).

**Figure 3:**
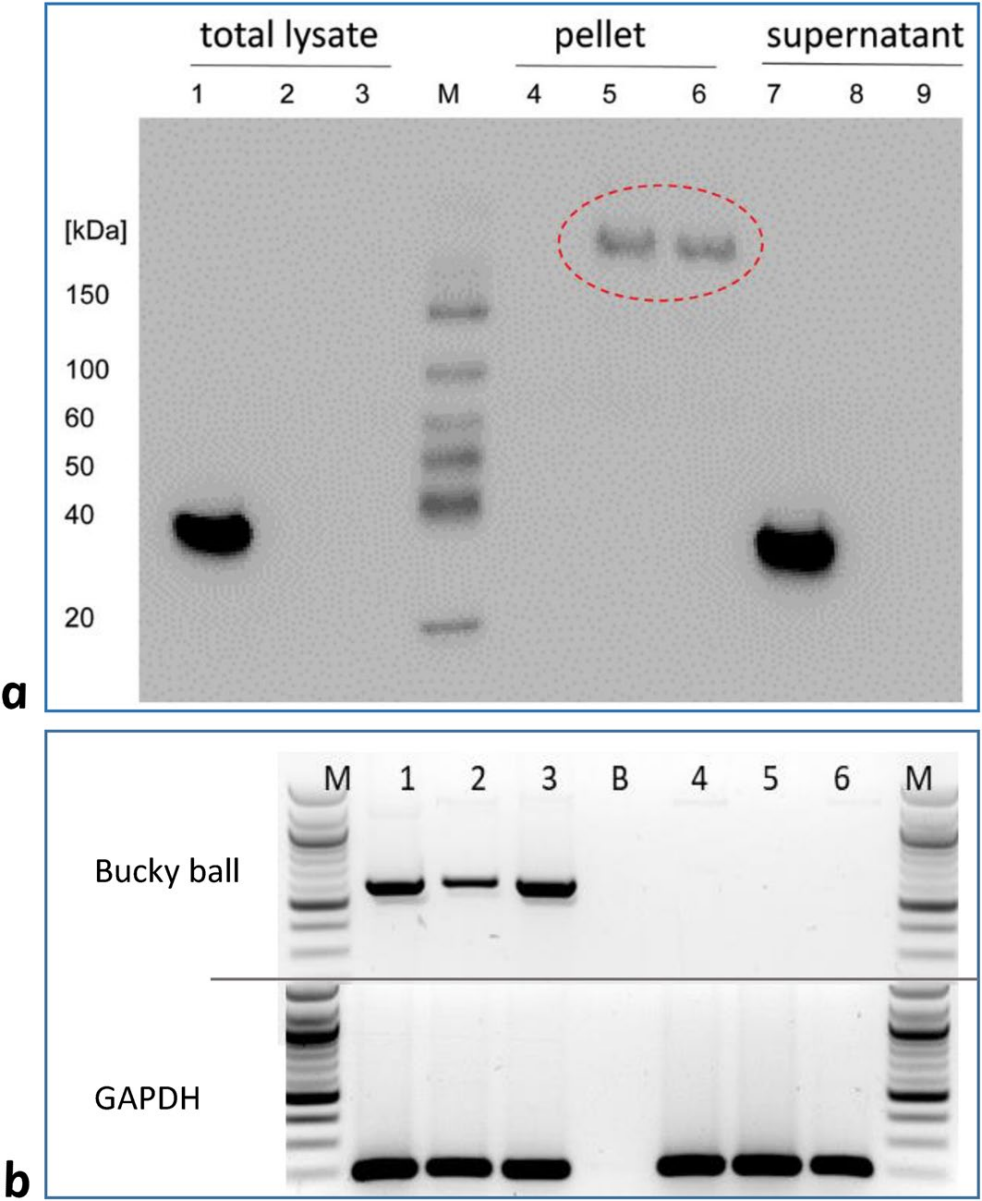
Expression of Bucky ball after transfection of fusion plasmids in vitro and in embryonic stages around oviposition in vivo **(a)** Bucky ball fusion-constructs form insoluble fusion proteins. Bucky ball fusion construct and controls (EGFP/Venus only) were transfected in DF1 cells, respectively. After 5 days, the cells were harvested, and total extracts, insoluble, and soluble fractions were assessed by Western blotting with an anti-GFP antibody. Controls (EGFP/Venus only, lanes 1, 4, and 7), zebrafish Bucky ball-EGFP (lanes 2, 5, and 8), and chicken Bucky ball-Venus fusion constructs. M, size marker with visible bands at 20, 40, 50, 60, 100, 150 kDalton. Note, the Bucky ball fusion proteins appear in the insoluble fraction (lanes 4, and 5) and migrate much slower than expected for the calculated molecular weights of 101 kDa (zBucky ball-EGFP) and 116 kDa (cBucky ball-Venus). The EGFP/Venus control shows a protein at the expected molecular weight of 27 kDa. (**b)** Detection of embryonically transcribed Bucky ball in the chicken blastoderm. The upper panel refers to Bucky ball and the lower panel to GAPDH transcripts. Samples 1 to 3 were generated from stage EGK X^28^ laid egg blastoderms, and samples 4 to 6 from 24 h incubated embryos (stage HH 6^27^). DNA-ladder in lanes (M): NEB 100 bp ladder N3231, (B) negative control amplification without template.

Next, the transfected cells were employed in Western blotting. Since the used anti-Buc antibody did not work in this assay, the blot was probed with an anti-GFP antibody. Therefore, total lysates, insoluble, and soluble fractions were loaded on a 10% SDS polyacrylamide gel (PAGE), blotted and incubated with the anti-GFP antibody. The results showed that the Bucky ball fusion proteins concentrate in the insoluble fraction and migrated poorly into the gel (Fig. 3a, lanes 5 and 6), most likely reflecting the liquid–liquid phase separation properties of IDP proteins.

As independent approach, and to check whether chicken Bucky ball fulfills the requirements of a germ plasm organizer, we employed the artificial intelligence (AI)-driven algorithm AlphaFold^26^ (https://www.alphafold.ebi.ac.uk). The protein structure prediction of well described germ plasm organizers from zebrafish (Bucky ball), xenopus (Velo1) and drosophila (Oskar) were downloaded from AlphaFold, and compared with the prediction for chicken LOC420748 (Fig. S9). AlphaFold calculates a per-residue confidence score (pLDDT) from 0 to 100, and pLDDT > 90 are expected to be modelled to high accuracy. Most regions/domains of chicken Bucky ball/LOC420748, zebrafish Bucky ball, Velo1 and to a lesser extent Oskar were assessed with low pLDDT scores (< 50 to 70), and the present structure predictions should be treated with caution. However, Fig. S9 highlights the stunningly similar unstructured nature of this molecule class, and strongly suggests that AlphaFold may be used to search for disordered proteins. Despite little or no sequence homology, all predicted protein structures of germ plasm organizers share the commonality of intrinsically disordered proteins, a feature most likely required for their interaction with different binding partners during germ cell specification.

### Expression of chicken Bucky ball mRNA

The expression of Bucky ball mRNA was investigated in three individual blastoderms of stage EGK X (after oviposition) in Fig. 3b, lane 1–3, and after 24 h of incubation (stages HH 5/6) in Fig. 3b, lane 4–6, respectively. Embryonically transcribed Bucky ball mRNA was unequivocally detected in all of the stage EGK X blastoderms, however no specific product could be amplified from the head fold embryos after 24 h of incubation (HH 5/6^27^).

### Binding of antibodies raised against zebrafish Buc

Intrauterine development of chicken was characterized in detail and classified in a normal table by Eyal-Giladi and Kochav in 1976 and 1980^28,29^. Therein, two principal phases of intrauterine development had been classified. Firstly, the cleavage phase with furrow cleavages from the center to the periphery of the germ until the definition of finally completely surrounded cells (stage I to VI). This phase is characterized by rapid incomplete furrow cleavages from the center to the periphery, stepwise, with successive closure of the cells at the ventral side. The second phase is described as area pellucida formation, with a severely reduced speed for cell divisions^29^. Area pellucida formation is mainly characterized by cell shedding from the center to reduce the around 5–6 cell layer thick blastoderm to a central epiblast layer (area pellucida) only one cell layer thick surrounded by the area opaca with four cell layers.

Germ plasm is known to be concentrated in the central part of the developing blastoderm from studies investigating germ cell markers^6,9,24^ and the evaluation of immunohistochemical labeling was concentrated in this region with comparative imaging of lateral frames for specificity controls (Fig. 7).

The specificity of two antibodies raised against zebrafish Bucky ball was investigated at stage EGK X chicken blastoderms firstly. Control samples without primary antibody and the complete set of secondary antibodies applied in the experiments respectively, were run in parallel in each immunohistochemical analysis and did not show specific labeling (Supplementary Fig. S5A). The isotype rabbit IgG control did not show any labeling either (Fig. S5B).

An antibody against pan-cadherin, was used as internal control verifying the Bucky ball specific labeling for subsets of cells stage EGK I, VII and X embryos (Fig. S6). The discrimination and independent labeling of the Bucky ball and pan-cadherin antibodies is demonstrated in Supplementary Fig. S6a in an embryo of EGK I/II, and in contrast to the selective labeling of Bucky ball in a limited number of cells in the center of the epiblast of stage EGK VII (Supplementary Fig. S6B2) and stage EGK X embryos (Supplementary Fig. S6D2). The selected pan-cadherin antibody labeled all of the membranes, but additionally the nucleus of blastodermal cells (abcam ab6529 product datasheet image 3 and 4, anonymous abreview, Supplementary Fig. S6B3, C2). This antibody specific effect was previously reported in other cell types and led eventually to an exchange of this antibody with increased specificity for the cell membranes. Nevertheless, as expected, the IgG rabbit raised Bucky ball antibody provided restricted labeling of clearly centrally localized cells, whereas pan-cadherin labeled the cell membranes of all cells, especially for the stages with clearly defined cells at stage EGK VII and X, characterized by cytoplasmatic Bucky ball immunoreactivity.

The total number of all controls are listed in Supplementary Table S2. No differences in the labeling pattern was observed between the two Bucky ball antibodies.

### Spatial–temporal expression of chicken bucky ball and co-localization pattern to the germline marker CVH

#### Oocyte to stage EGK II embryos

**Figure 4:**
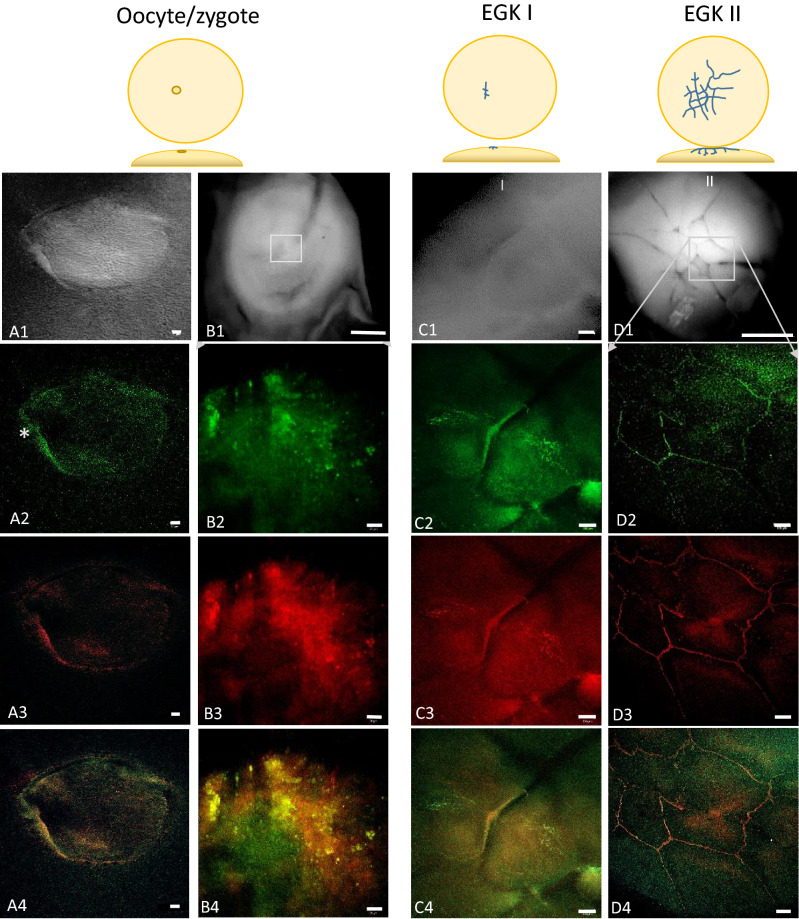
Accumulation of Bucky ball protein from the oocyte to early cleavage stages (EGK I/II) in chicken embryos. The upper panel represents differential interference (**A1**) and bright field images (**B1,C1,D1**) of the germinal vesicle (**A1**), the total fragment containing the germinal disc (**B1**) and the germinal disc regions (**C1,D1**). Frames indicate the region of the higher magnified fluorescent images in the lower panels. Freshly ovulated oocytes are characterized by a diffuse distribution of the Bucky ball protein from a condensed outline of the germinal vesicle (**A2**). At the outline, a higher Bucky ball concentration at one side (*) is apparent. After zygote formation, when the fertilized oocyte reached the isthmus part of the oviduct 2 to 2.5 h after fertilization, Bucky ball protein concentrates in granular patches in the center of the germinal disc where first cleavage furrows will be formed (**B2**). The first cleavage furrow is lined by tightly aggregated Bucky ball, and in perpendicular orientation, the Bucky ball granules demarcate the predicted next furrow (**C2**). During the next furrow divisions of stage I and II, further Bucky ball protein condenses in the cleavage furrows in the center of the forming embryo (**D2**). There is no clear demarcation of cellular outlines yet. This pattern of protein distribution is highly colocalizing to CVH (**A3–C3**), one of the first known germ plasm proteins as visualized in the overlay in **A4–C4**). Scales represent for (**A**) = 20 µm, (**B1**) = 200 µm; (**C**) = 50 µm; (**D1**) = 200 µm, the panels (**B2–B4**) = 20 µm, and (**D2–D4**) = 50 µm.

**Figure 5:**
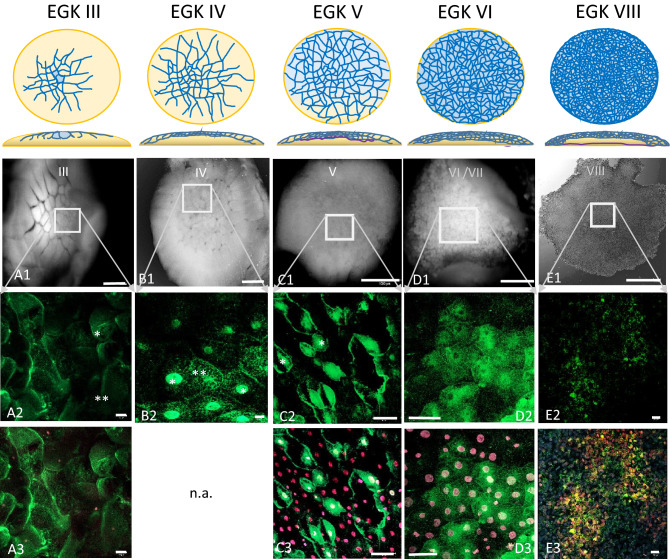
Dynamic localization of Bucky ball around major zygotic genome activation. The upper panel represents schemes for the formation of cells from stage EGK III, where first centrally located cells get separated to stage VIII, the second stage of area pellucida formation phase as described by Eyal-Giladi and Kochav^28,29^ (**A1**) An eccentrical position (rectangular frame in (**A1**), upper left panel) of the cleavage center within the chicken germinal disc region. With the ongoing cell divisions during EGK III, Bucky ball protein granules start to accumulate in the cytoplasm of the medial cleavage center in first laterally and ventrally closed cells (* in **A2**). The protein is not found accumulating around or in the nuclei stained with SIR and visualized in red. In the more lateral cells, the protein is still located in or around the cleavage furrows (**). At seven to eight hours after fertilization (hpf) during stage EGK IV, Bucky ball is first observed in the nuclei of central cells with very high intensity of labeling (B2*), whereas at the surrounding cells Bucky ball is still located in the cytoplasm (B2**). The same pattern of intracellular protein localization of Bucky ball was recorded in stage V embryos (**C1** overview, **C2** magnified central frame with nuclear and cytoplasmic labeling of Bucky ball. (**A3**,**C3–E3**) demonstrate SIR counterstained nuclei and the concentration of Bucky ball in and around them. After around 12 hpf, when embryos developed to stage VI, Bucky ball seems to decrease in intensity overall and the nuclear signals disappear (**D2**). This process of signal reduction proceeds further to stage VIII and no nuclear labeling remained (**E2**). The basal membrane is separating the yolk from the developing embryo, and starts growing from the middle to the edges of the disc at stage V, (purple line, scheme above column **C**) and reaches the outline of the disc at stage VI (scheme, column **D**). The subgerminal cavity is formed between the embryo and the basal membrane via cell shedding to the outline forming the area pellucida from stage VII onwards (scheme, column (**E**)). Scales (**A1–E1**) = 1 mm, (**A2,A3,C2,C3**) = 50 µm; (**B2,D2,D3**) = 10 µm, (**E2,E3**) = 20 µm.

**Figure 6:**
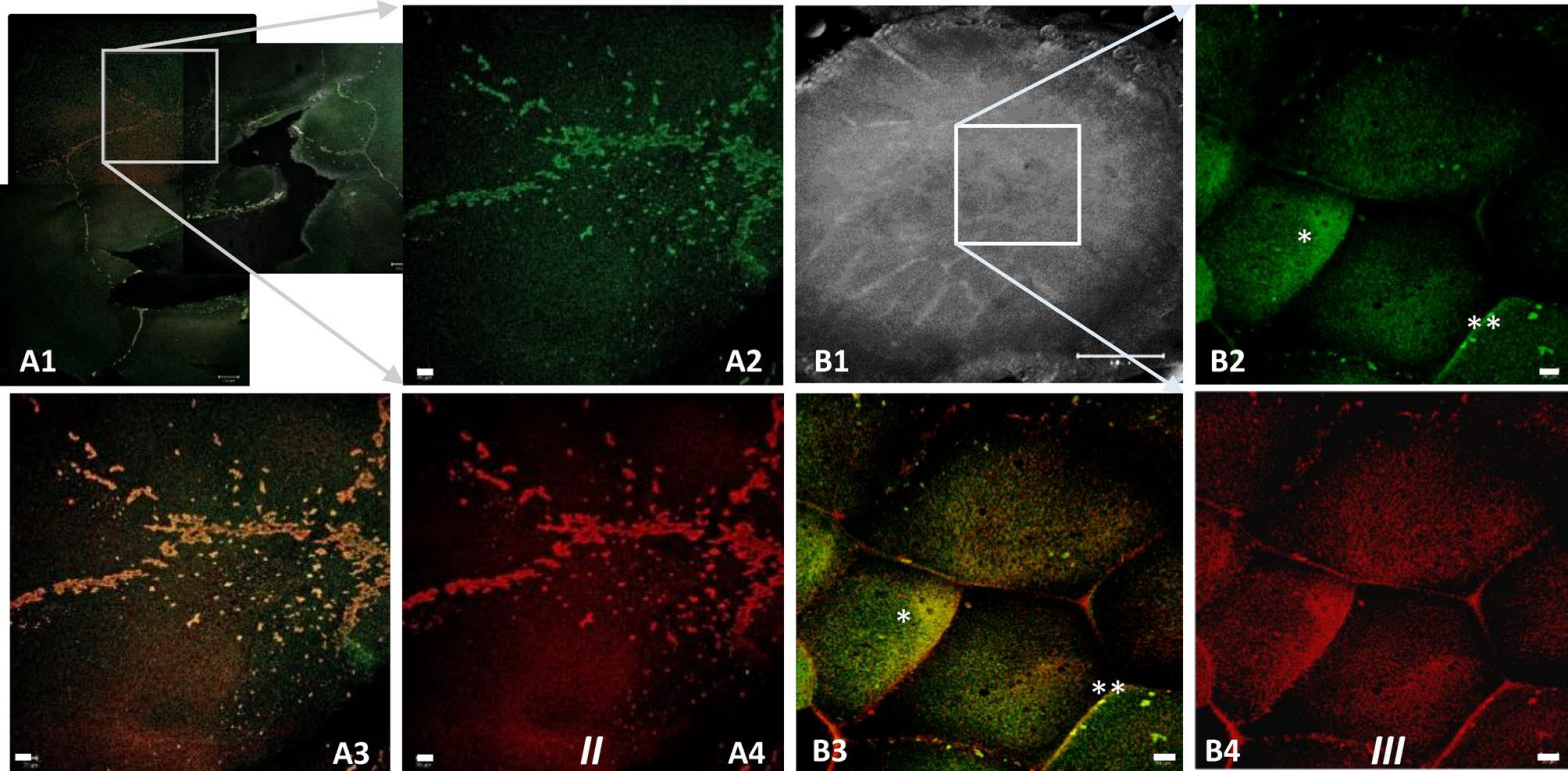
Co-localization of Bucky ball and CVH during stages EK I to EK III. During the first two cleavage stages (EK I and EK III), Bucky ball strictly co-localized to CVH within the cleavage furrows and in condensed granules in the cleavage center A: (**A1**) stitched fluorescent overlay images of the central region of an EGK II embryo with the indicated region of high resolution images shown in (**A2–A4**) (**A2**) Bucky ball, (**A3**) CVH, (**A4**) overlay. (**A2—A4**) are included in a preprint^51^. During the next stage, EK III, both germ plasm proteins translocate into the cytoplasm of the first forming central cells B: (**B1**) transmission image of the total embryonic disc at stage EGK III with indicated central region for high resolution fluorescent imaging: (**B2**) Bucky ball, (**B3**) CVH, (**B4**) overlay. Asterisk indicate the diffusion of Bucky ball and CVH proteins into the cytoplasm. Scales 20 µm, except (**B1**) = 500 µm.

Dispersed maternal Bucky ball proteins were found in matured oocytes (Fig. 4A2) and start to aggregate in the zygote around the site of the first cleavage furrows (Fig. 4B2). This pattern of pre-aggregation of granular spots occurs in the zygote around the regions of cleavage furrow formation (Fig. 4B2). Importantly, the Bucky ball signals co-localized with the pattern of CVH labeling, yielding in an accumulation and further condensation of both proteins around and within the cleavage furrows during stage EGK I to EGK III (^1^, Figs. 4C2–D4, 5A2—A4, B2–B4). A very high proportion of cells co-localized Bucky ball protein and CVH in the same cells in all embryonic stages investigated (Fig. 3, and Figs. 5, 6, 8 and 9).

Cleavage furrow localization of Bucky ball and CVH extends throughout stage EGK I and EGK II, distributing germ plasm into centrally located 8–16 progenitor cells of the maximum 64, partly laterally and ventrally open cells at the end of stage EGK II. At this time, the yolk is surrounded by the first albumin layer, has passed the isthmus of the oviduct and entered the uterus.

**Figure 7:**
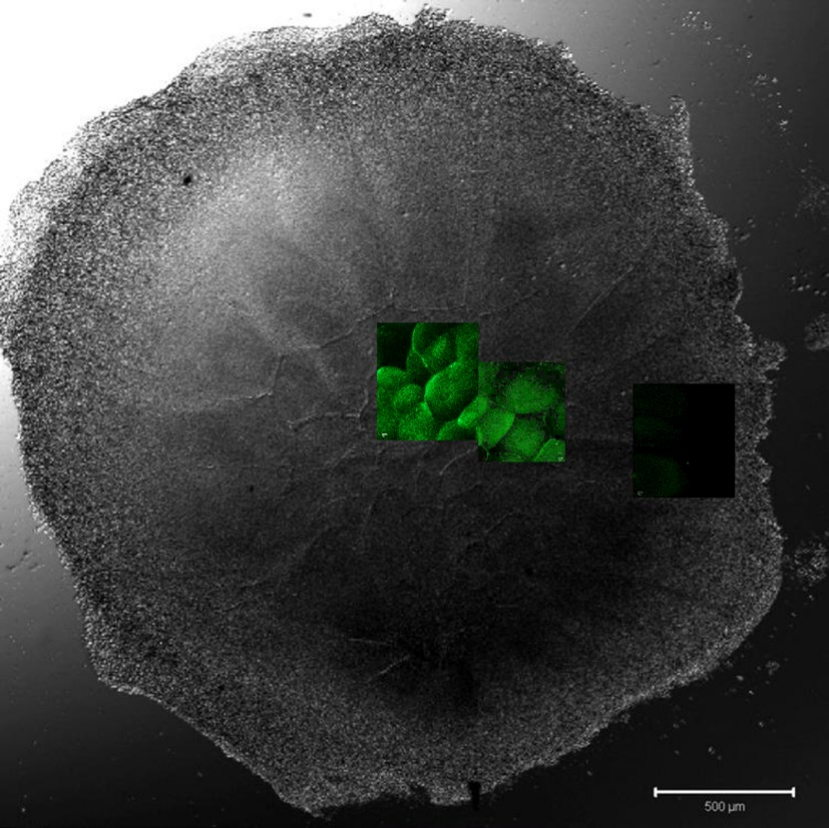
Overview of an intrauterine embryo (EGK III) with two frames of the central region with Bucky ball immunolabeling in contrast to severely reduced immuno-fluorescence at the lateral outline of furrows. The two frames in the central region show the cells with Bucky ball immunofluorescence in cytoplasmic granular distribution. The lateral frame, documented at the exactly comparable acquisition conditions, does not provide signals above background. Scale = 500 µm.

**Figure 8:**
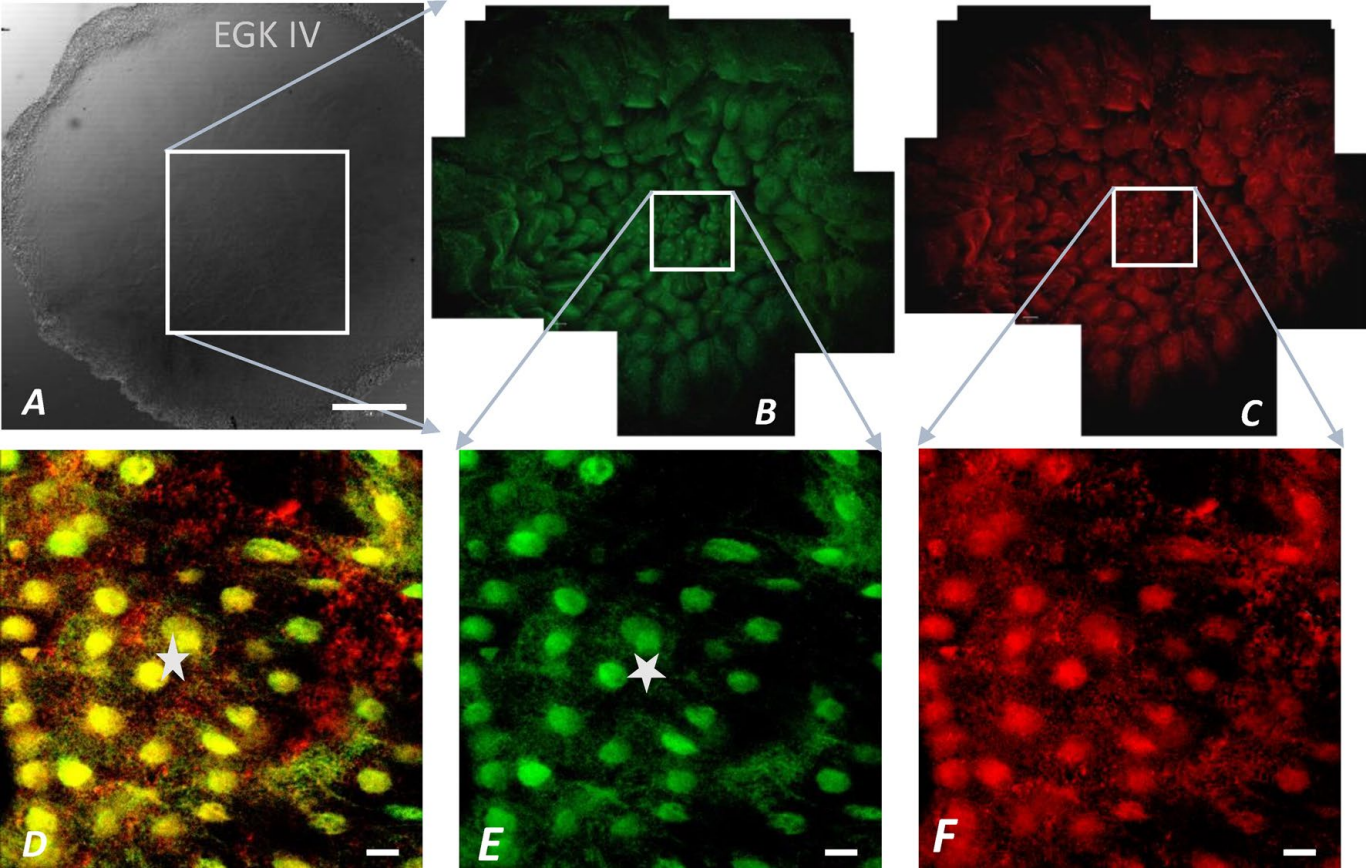
Co-localized Bucky ball and CVH expression in stage EGK IV. This composition of eight stitched images visualizes in the magnified center of a stage EGK IV embryo (frame of the overview in (**A**) the concentrated nuclear localization of Bucky ball (**B**) and CVH (**C**) within the central cells of the germinal disc surrounded by cells with cytoplasmic germ plasm of Bucky ball and CVH. In (**D**) the overlay with clear co-localization of Bucky ball (**E**) and CVH (**F**) in cells of the nuclear region of EGK IV embryos is shown. Numbers with nuclear region labels exceed 20 cells repeatedly. Scale in (**A**): 500 µm, scales (**D–F**): 20 µm.

**Figure 9:**
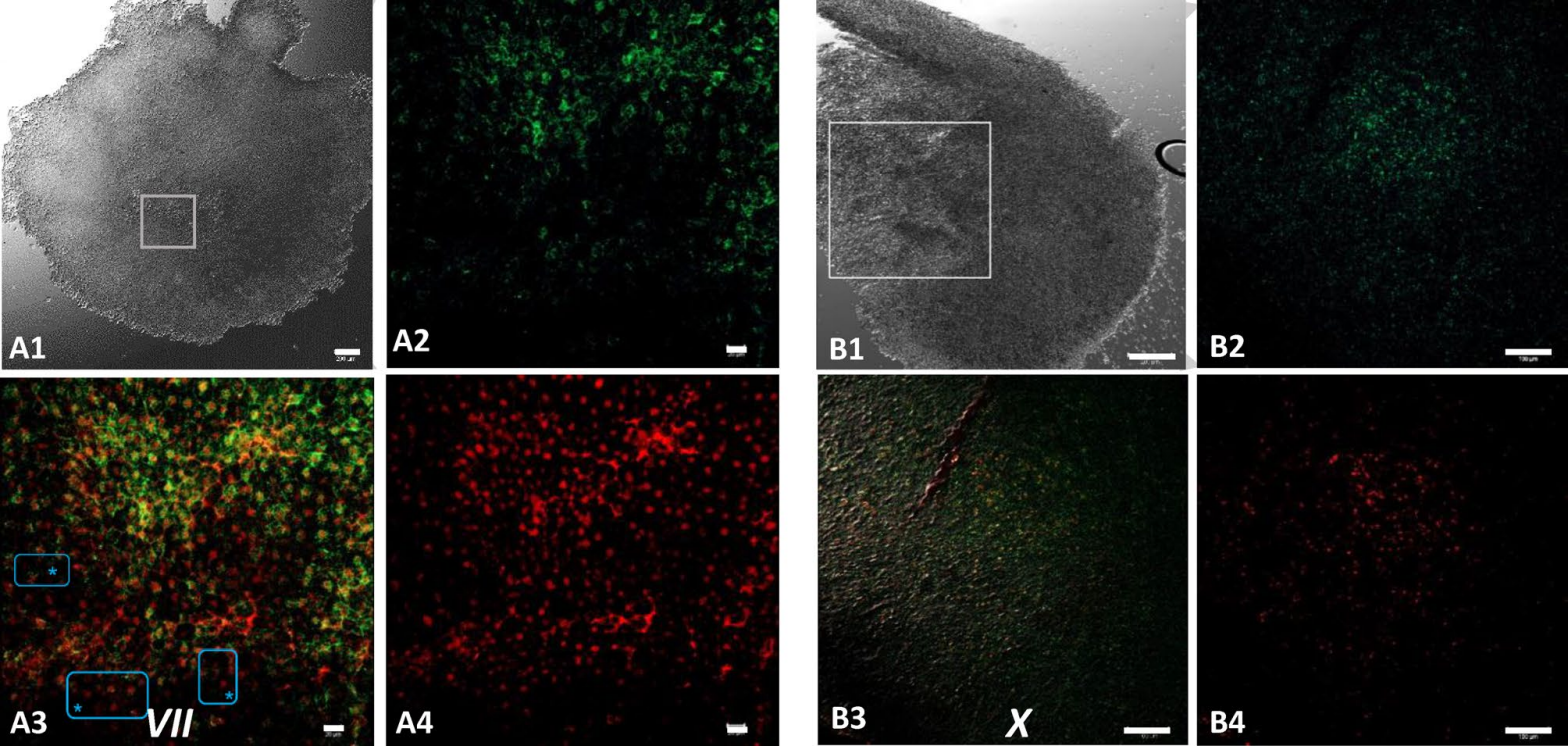
Bucky ball and CVH are no longer co-localized within the nuclear region of primordial germ cells during the *area pellucida* formation. 7A, in EGK VII embryos, at the beginning of area pellucida formation, Bucky ball (green) in the center of the germinal disc diffused back into the cytoplasm (**A2**), while CVH (red, **A4**) is contained predominantly in the nuclei. This is visualized clearly in the overlay (**A3**) as a red center of the germline predisposed cells surrounded by the green Bucky ball fluorescence. Blue frames with asterisk indicate only CVH labeled without Bucky ball at the lateral edges of the central region. DAPI as nuclear marker visualizes the central concentration of the germline cells and as white overlay the nuclear localization of CVH and DAPI in the germline cells. This pattern is stable until oviposition at stage X (**B**). Scales in (**A1**) = 200 µm, (**B1**): 500 µm, (**A2–A4**) 20 µm, (**B2–B4**): 100 µm.

Interestingly, a dynamic intracellular distribution of the two germline-specific proteins was observed during stage EGK III to VIII of intrauterine development. (Figs. 5, 6, 7, 8 and 9).

### Embryos from EGK III to VI

As cell divisions progress (EGK III), both Bucky ball as well as CVH protein granules begin to accumulate in the cytoplasm of the medial cleavage center in the first completely closed cells (Figs. 5A2; 6B2–C2). In the more lateral cells, the proteins still locate in or around the lateral open cleavage furrows (Figs. 5A2**, 6A2). During these early cleavages, Bucky ball and CVH are concentrated in the central part of the germinal disc in the cells that have just fully closed (Fig. 5). Bucky ball protein is shifted further from the cytoplasm in stage EGK III embryos (Fig. 5A2) into the nuclei of the most central cells in stage EGK IV (Figs. 5A2, B2, 6, 7). This nuclear translocation is observed in parallel and with consistently high degree of co-localization for CVH (Fig. 8). The nuclear localization of Bucky ball was observed in stage EGK IV and V embryos consistently in the central cells (Figs. 5B2,C2, 8). Interestingly, Bucky ball starts to diffuse back into the cytoplasm at stage EGK VI (Fig. 5D2), whereas CVH stays predominantly localized in the nucleus throughout the formation of the *Area pellucida* (Fig. 9).

### Stage VII to stage X

After the onset of *area pellucida* formation, the distribution of Bucky ball and CVH separated within the germline predetermined cells. Bucky ball moved into the cytoplasm of the cells as shown in an embryo of stage EGK VII (Fig. 9A2), whereas CVH consistently labeled the nuclei of the germline cells (stage VII, Fig. 9A4). This separated localization of Bucky ball and CVH within the presumed germline cells was found during further development to stage EGK X (Fig. 9B).

The number of cells containing Bucky ball steadily increased rapidly until stage EGK V to about 50 cells. Thereafter, there was no significant further increase in primordial germ cell number seen until oviposition.

At oviposition, the blastoderm is composed of around 60.000 cells. Remarkably, the labeling for CVH (Fig. 9A4,B4) seems to exceed the number of cells labeled for Bucky ball at these stages laterally (Fig. 9A3, blue frames and * and B3). A strong intensity gradient from highest protein concentrations in the central cells to very low protein labeling at the edge of the central region at the upper right corner of Fig. 9A, and to no detectable protein labeling in the outer third of the blastoderm at the lower left corner was most impressive at these stages (Fig. 9B2,B4).

## Discussion

In this study, the currently uncharacterized chicken sequence LOC420748 was experimentally investigated. The experimental data provided in this manuscript strongly support the notion that the unannotated chicken LOC420748 encoded a functional equivalent of the zebrafish germ plasm organizer Bucky ball, despite a lacking homology at the nucleotide level. By protein BLAST two conserved peptide domains could be identified between LOC420748 and zebrafish Bucky ball: a N-terminal domain of 134 amino acids and a central domain of about 100 amino acids^11,12^.

We demonstrated the functional properties of this protein with several independent lines of evidence.

Firstly, the fusion proteins composed of the chicken and zebrafish cDNA sequences for Bucky ball and fluorescent reporters, respectively, verified a comparable condensation pattern for the expressed proteins in three different chicken cell lines. The fusion protein condensation resemble typical features of liquid–liquid phase separation of IDPs. Secondly, western blotting confirmed the insoluble character of chicken and zebrafish fusion proteins, apparently both migrated much slower into the gel than predicted from the calculated molecular weight. Thirdly, the amino acid sequence analyzed with the AlphaFold Protein Structure Database predict for the chicken LOC 420478 a thoroughly disordered structure as characteristic for Bucky ball in zebrafish, Velo1 in xenopus and Oskar in drosophila (Supplementary Fig. S9).

Moreover, transcription products of chicken Bucky ball were recorded in blastoderms at oviposition, but not during gastrulation later than 24 h of incubation (Fig. 3b). Thus, the expression is limited to the intrauterine phase of development when specification of primordial germ cells is achieved.

And last but not least, a dynamic pattern of protein distribution was observed by immuno-histochemical labeling of intrauterine embryos. In oocytes, a dispersed pattern of chicken Bucky ball was found, followed by an aggregation in cleavage furrows during the first two cleavage stages. At stage EGK IV to EGK VI, the time of the second zygotic genome activation wave, a clear nuclear labeling of Bucky ball was evident in the central cells that are at first completely surrounded by cell membranes. In these stages, a strong co-localization with CVH in the nuclei of the presumptive primordial germ cells is obvious. The number of cells with bucky ball and CVH in the central cells from stage EGKV onwards was higher than 15 cells and lower than 60 cells in all samples recorded. This is agreement to data recorded by Lee et al.^24^, who reported 50–80 cells positive for the germline marker Dazl-between stage EGKV and VIII.

The rapid increase of germline cells during EGK I–IV and fairly stable number of germline cells during the area pellucida formation reflect a slowing down in cell proliferation as already reported for the total cell numbers (stage EGK VII to X)^6,29^. Remarkably, a perinuclear co-localization of Bucky ball and Vasa was reported during the period of PGC specification in zebrafish^13^. Thus, the nuclear and perinuclear localization of Bucky ball and CVH during the second and major wave of zygotic activation is suggested of special importance for the specification of PGCs in chicken.

During the subsequent cellular reorganization within the chicken blastoderm, the area pellucida formation, Bucky ball was shifted back to the cytoplasm of the PGCs, whereas CVH stayed in the nuclei of PGCs at least up to 48 h of incubation.

Remarkably, the evolutionary conserved functional parameters of liquid–liquid phase separation, characteristic for the IDPs with germ plasm organizer functionality like Oskar from *Drosophila*, Bucky ball in zebrafish, and Velo1 in Xenopus^10–12,30,31^, is based on very limited amino acid homology of the proteins. In zebrafish, strings of approximately 100 amino acids were found to be responsible for the localization of Bucky ball in the germ plasm and the binding of RNA of several established germ plasm proteins as VASA and NANOS mRNA^11,12^. Here, we show that these short peptide sequences are conserved between zebrafish and chicken. Before, homology in these domains between zebrafish and other species, such as Xenopus^31^^,^^32^ have been reported. The report of Perera et al. further specified the vasa-binding motif in zebrafish (Fig. 1 in^18^) to a shorter 23 amino acid sequence. Remarkably, this short motif matches 75% of chicken Bucky ball amino acid sequence (Fig. 1b) and 80% of the respective sequence in Xenopus^12^.

In the chicken, a distribution pattern of germ plasm is observed for Bucky ball with strong co-localization to CVH in the cleavage furrows of the first two stages. This pattern was very similar to the localization of Bucky ball in zebrafish^11,13^, but the distribution of germ plasm is not restricted to the first two cleavages and results in many more germline cells.

At stage EGK III, the first cells readily surrounded with cell membranes contain the germ plasm marker CVH together with Bucky ball in the cytoplasm. These three to eight cells continue to divide and the number of cells containing germ plasm markers increases steadily to about 50 PGCs in the blastoderm at oviposition^33,34^. There was no evidence of uneven distribution of germ plasm among daughter cells as the number of Bucky ball positive cells increased continuously. This is in contrast to the specification of germline in zebrafish, where after two cell divisions from zygote formation the first four cells cover the germ plasm, and further unequal cell divisions ensure the consistency of the germ plasm in only four cells until the 1000 cells stage when gastrulation starts^13,35,36^. Whereas germ plasm inherited germline definition follows a common strategy, the cell numbers required for germline manifestation, their localization within the early embryo, and the time line for specification of cell lines are differently resolved between zebrafish, *xenopus* and chicken^37^.

In chicken, a first wave of mRNA expression is reported as minor wave of zygotic activation from oocyte to zygote formation^38^. Stage EGK III is referred to as the onset of the major maternal to embryo transcription (MET), indicated by the appearance of visible nucleoli^39^ and the first time point of nuclear localization of polymerase II and maternal specific zygotic transcription^38,40,41^. The rapid increase in zygotic transcription is highest between EGK IV to VI and coincides with the nuclear localization of the Bucky ball immuno-labeling reported here.

Finally, during the second half of intrauterine development, during cellular reorganization into the blastoderm, named area pellucida formation, chicken Bucky ball is again released from the nucleus into the cytoplasm, while CVH is continuously located within the nucleus.

A screen for oocyte-specific genes^42^ described the exclusive expression of *CVH, WEE, ZAR1, DAZL, ZP-A*, and *BTG4* in early cleavage embryos. For both germline markers *DAZL*^24^ and *CDH*^43^, the distribution pattern of protein in oocytes was demonstrated as part of the germ plasm with cleavage furrow localization during the first two stages of cleavage. Thus, chicken Bucky ball co-localizes with several known germ plasm markers in the matured oocyte and the first two stages in the cleavage furrows of the still ventrally and lateral open cells of the telolecithal embryo (Figs. 4A2–D2, 5A). However, the functional network for restriction of the germline functional proteins to predetermined PGCs and the eradication of germline signaling in somatic cells remains to be elucidated. The position of Bucky ball in this transcriptional network needs further investigations.

Here, we show data that the expression of Bucky ball is limited to the early development in chicken. These data confirmed a previous transcription analysis describing expression of the unannotated LOC420748 in laid egg blastoderms, but neither after 12, 24 nor 30 h of incubation^44^.

The first reports of molecular germ line markers indicated that the chicken germ plasm is assembled during oogenesis, as in zebrafish and *Xenopus,* and indicate that PGCs are specified before gastrulation^38,40–42,45,46^. Thus, chicken PGCs could be referred as the first specified uni-potent cell lineage already formed in the laid egg blastoderm. Lavial^47^ experimentally linked quantitative differences in *CVH* expression of EGK X blastodermal cells to differences in the expression of further germline and germline inductive markers as *DAZL, POUV, SDF1, TUDOR* and *NANOG*. During the first two cell divisions, DAZL could potentially lag behind in the reorganization of the CVH protein granules from the dispersed state in the oocyte to a concentration in the cleavage furrows as compared with CVH^6^. However, during stage EGK III, the common cytoplasmic localization in the central cells of the cleavage embryo is consistent for CVH and DAZL. Thus, the first established cells express both markers.

Here we demonstrate in chicken, that maternally provided Bucky ball is present in matured oocytes and cleavage embryos as functional protein until the laid egg blastoderm. It is localized in a pattern consistent to a germ plasm organizer. Interestingly, the Bucky ball gene is highly conserved within the avian clade (Supplementary Fig. 7). This exceptionally high homology within birds could imply an extended functional importance for Bucky ball in birds.

Interestingly, a clearly nuclear labeling and co-localization of Bucky ball together with CVH, is restricted to the major activation of the zygotic genome in the central cells of the forming embryo during stage IV to VI. This suggests the major period of zygotic genome activation with nuclear co-localization of Bucky ball and CVH as important phase for germline specification, and suggests a germ plasm organizer function for Bucky ball in chicken. The presented findings warrant further studies to pinpoint the function of chicken Bucky ball especially during oogenesis.

## Materials and methods

### Protein sequence analysis

The NCBI BLAST tool (BLASTn and BLASTp) was used for homology comparisons (https://blast.ncbi.nlm.nih.gov/Blast.cgi). The cNLS Mapper (https://nls-mapper.iab.keio.ac.jp/cgi-bin/NLS_Mapper) was employed for prediction of nuclear localization signals (NLSs) in the amino acid sequence specific to the importin αβ pathway by calculating NLS scores^25^. The NLS scores are calculated with four NLS profiles (for class 1/2, class 3, class 4, and bipartite NLSs), each of which represents a contribution of every amino acid residue at every position within an NLS class to the entire NLS activity^25^**.**

For prediction of the protein structures of germ plasm organizers the AlphaFold Protein Structure Database (https://alphafold.ebi.ac.uk) was accessed^26^, and protein structure predictions of LOC420748, zebrafish Bucky ball, Velo1 and Oskar were downloaded.

### Recovery of ovarian follicles, and intrauterine stage cleavage embryos

Adult hens of commercial layer hybrids or hens from genetic resource lines of the Friedrich-Loeffler-Institut at 50 to 65 weeks of age, characterized by regular daily ovulations, were artificially inseminated weekly with a volume of 50–100 µl of fresh mixed semen from 2 to 4 roosters after macroscopic visual assessment of semen quality.

Prior to the start of the experiment, oviposition was monitored for two weeks and at hourly intervals for the last 5 days, to predict the expected next ovulation, which is assumed to occur approximately 15–30 min after the last oviposition.

The recovery of ovulated oocytes and matured follicles was realized after carbon dioxide exposure of the hens, in agreement to the German laws regulating animal welfare. The German legislation does not require an ethic approval for painless killing of animals for scientific purposes (Tierschutzgesetz, §4, Abs. 3).

The ovulated eggs were recovered from the oviduct, and the superficially accessible germinal disc regions (GD) were excised from the ova to remove the massive yolk, surrounding albumin, shell membranes, or shell depending on the stage of the embryo. The development of the fertilized oocytes and embryos was staged according to Eyal-Giladi and Kochav^28,29^.

Intrauterine stages were sampled at 17 different time points for a total of 14 immunocyto-chemical assays. Each reported stage was analyzed in at least three separate experiments and immunocytochemically analyzed comparative to one to four other stages (Supplementary Table S2).

### Fixation of germinal disc fragments as well as cells on cover slips and immunohistochemistry

The GDs were fixed in 4% formaldehyde in a 0.1 M PBS-solution (Supplementary Table S3) for about 3 h before the samples were transferred in 0.1 M PBS supplemented with 0.1% sodium azide for a maximum of 14 days.

Immunostaining was performed as whole mounts of the GDs in suspension^13,48^. Shortly, after six washes in 0.02 M PBS (Supplementary Table S3), a 2 h-blocking step was performed in 0.02% M PBS including 0.05% Triton X100 and 2% bovine serum albumin (BSA). The primary antibodies were diluted in 0.02 M PBS, 0.1% BSA, 0.1% NaN_3_, 0.05% Triton X100.

The Bucky ball antibodies were used both at a 1:3000 dilution (antibodies were raised in guinea pig and rabbit against the zebrafish Bucky ball full length protein (BioGenes, Berlin), rabbit anti-pan-cadherin (abcam ab6529) 3.3 µg/ml, and rabbit anti-CVH (Bioss, bs-3597R) at 5 µg/ml in different combinations. Incubation was performed for 44–48 h at 4 °C. After several washes in 0.02 M PBS (Supplementary Table S2), the secondary antibodies were applied for 3 h (goat anti-rb-Alexa Fluor 555, Thermo Fisher A-21430, 2 µg/ml; anti-guinea pig-FITC (Vector lab. FI-7000, 1.5 µg/ml). After washing, the tissue fragments were mounted on slides with Vectashield mounting medium (Vector lab. H1000) including either SIR-DNA (1 µM, Spirochrome) or DAPI (2 µg/ml, SIGMA D9542) as nuclear counterstain.

The specificity of binding was previously tested on blastoderm samples from laid eggs. After adjustment of each single antibody concentration, the combination of primary and secondary antibodies was tested. The combined use of antibodies against pan-cadherin and Bucky ball was applied to verify specificity of binding in embryos with completely formed cells of stage EGKIII–EGKX. For validation of specificity of binding, each immunohistochemical procedure contained negative control samples (samples without primary antibodies and complete panel of secondary antibodies (Supplementary Fig. S5A), Additionally, an isotype rabbit-IgG control was tested (Supplementary Fig. S5B).

### Confocal imaging

A laser point-scanning laser confocal microscope (LSM510) was used to detect the fluorescence signals using the AIM-software (Carl Zeiss Microscopy, Jena). Single channel excitation in multiplex mode was applied to separate the different fluorescence labels.

### Construction of bucky ball-reporter fusion plasmids

The previously described zebrafish Bucky ball cDNA fused in-frame with an EGFP-sequence driven by a cytomegalovirus (CMV) promoter was used (pCS2-buc-eGFP^12^). The chicken Bucky ball-cDNA sequence was isolated by a RT-PCR approach from chicken intrauterine embryos. Thereafter, the cDNA without a stop codon was fused with a Venus sequence (pT2RMCE-CAGGS-Venus) by a PCR-based approach with chimeric primers, resulting in pT2RMCE-cBuckyball-Venus. The pCS2-buc-eGFP contains a chimeric CMV enhancer and beta actin (CAGGS) promoter for ubiquitous expression.

### Cell culture and transfection of cells

DF-1 cells were received from the cell bank of the Friedrich-Loeffler-Institut and cultured in DMEM medium supplemented with 2 mM L-glutamine, 0.1 mM mercaptoethanol, 0.4 mM sodium pyruvate, non-essential amino acids, 10% fetal calf serum, and 1× penicillin/streptomycin (D10). Chicken primary fibroblasts were derived from day 10 (E10) embryos and maintained in D10 medium.

PGCs were derived from chicken embryonic gonads (E7, stage HH 28–29) and cultured as described^49^. The PGCs were cultured in suspension without a feeder-layer in customized avian KO-DMEM (23). Shortly, a CaCl_2_-free medium (12.0 mM glucose, 250 mOsm) produced by ThermoFisher-scientific was used as the basal medium. It was supplemented with 1× B-27, 2.0 mM GlutaMax, 1× NEAA, 0.1 mM ß-mercaptoethanol, 1× nucleosides, 0.4 mM pyruvate, 0.2% ovalbumin (Sigma), 0.1 mg/ml sodium heparin (Sigma), 0.15 mM calcium chloride, 12.5 ng/ml human activin A (PeproTech), 4 ng/ml basic fibroblast growth factor (PeproTech), and 0.2% chicken serum (ThermoFisher scientific). All cells were cultured at 37 °C and 5% CO_2_. For the transfections, total 1.5 × 10^6^ cells were suspended in 200 µl R-buffer with 10 µg plasmid-DNA. For transfections, the Neon Transfection System (ThermoFisher scientific) was used (1700 V, 20 ms, 1 pulse).

### Western blot analysis of bucky ball-fusion construct transfections

PGCs, primary chicken embryonic fibroblasts, and DF1 cells were transfected with the zebrafish Bucky ball-EGFP, chicken Bucky ball-Venus, and Venus only (control) constructs, respectively. Five days after transfection, the cells were washed and lysed in radioimmunoprecipitation buffer (RIPA, 500 µl per well of a 6-well plate) for 30 min on ice as described^50^. Half of the RIPA extracts were fractionated into a pellet and a supernatant fraction by centrifugation at 14,000 rpm. The pellets were directly resuspended in Laemmli loading buffer, 10 µg of total extracts, pellet, and supernatant fractions were loaded on a 10% SDS-PAGE, separated, and blotted to a polyvinylidene difluoride (PVDF) membrane. After blocking with 5% non-fat milk powder, the PVDF membrane was probed with a mouse anti-GFP monoclonal antibody (Developmental Studies Hybridoma Bank (DHSB), #86/8, 1:500). Bound primary antibodies were detected via a horseradish peroxidase–coupled secondary antibody (1:10,000; Sigma) and an enhanced chemiluminescence reagent (Westar Supernova, Cynagen, #XLS3). The Venus fluorophore is a derivative of the EGFP with only three amino acid^50^ exchanges, and thus cross reacts with most anti-GFP antibodies^50^. The resulting Western blot data were identical for all three cell types.

### RNA-extraction and RT-PCR

Blastoderm- and head-process stage embryos were dissected from the yolk and rapidly frozen at a temperature of − 80 °C. Total RNA from samples were extracted using 500 µl Trizol reagent (ThermoFisher-scientific) and 100 µl chloroform, washed with 250 µl isopropanol and twice with 70% ethanol. After drying, the pellet was resolved in nuclease-free water and 1 µg RNA was reverse transcribed (QuantiTect Reverse Transcription Kit, Qiagen) including oligo-dT and random primers following manufacturer’s instructions.

The PCR conditions were set as: initial denaturation at 95 °C for 2 min, and 35 cycles of 94 °C for 30 s, 60 °C for 60 s, 72 °C for 30 s using 50 ng cDNA in case of chicken Bucky ball. In case of GAPDH, 20 ng cDNA were used as template for 35 cycles. Final extension of 72 °C for 5 min each was added. The reaction products were resolved using a 1.5% ultrapure agarose (Invitrogen) gel electrophoresis, and visualized at a trans-illuminator. Intron-spanning primers are listed in (Supplementary Table S4).

## Supplementary Information

Supplementary Information.

## Abbreviations

CVH: Chicken vasa homolog
CEF: Chicken embryonic fibroblasts
DAZL: Deleted in azoospermia-like
EGK (I-X): Definition of intrauterine stage differentiation according to the normal table of Eyal-Giladi and Kochav
EMA1: Primordial germ cell surface marker (mouse) is a monoclonal IgM antibody available from the Developmental Studies Hybridoma Bank (AB_531885), and recognizes primordial germ cells in a wide variety of species with minor epithelial cross reactivity
HH: Definition of stages for chicken embryonic development during incubation according to the normal table of Hamburger and Hamilton
IDP: Intrinsically disordered protein
nts: Nucleotides
NLS: Nucleus localization signal
PGCs: Primordial germ cell
SSEA1: Stage-specific embryonic antigen 1 is a monoclonal IgM-antibody available from the Developmental Studies Hybridoma Bank (MC-480) and recognizes early embryonic stem cells and primordial stem cells in mammals and birds
