# Supplementary material for: Identification of chicken LOC420478 as Bucky ball equivalent and potential germ plasm organizer in birds
Source: Sci Rep. 2022 Oct 7;12:16858. doi: 10.1038/s41598-022-21239-8 (PMC9546911; doi:10.1038/s41598-022-21239-8)
Supplement: Supplementary file 1 — Supplementary Information. [file 41598_2022_21239_MOESM1_ESM.pdf]

Identification of chicken LOC420478 as Bucky ball equivalent and potential germ plasm organizer in birds

Sabine Klein[#1], Roland Dosch*, Stefanie Altgilbers[#], Wilfried A. Kues[#]

List of supplementary data

Fig. S1: Transcript XM 040690219 of LOC 420748

Fig. S2: Amino acid sequences of the predicted chicken (left panel, chicken protein-id=XP_ 040518588.1) and zebrafish Bucky ball genes (right panel)

Fig. S3: Plasmid map of the chicken Loc 420748 cDNA without stop codon fused to Venus

Fig. S4: Expression of fusion constructs of Bucky ball and green fluorescent protein reporters in different chicken cell lines.

Fig. S5: Specificity controls without primary antibody and anti-rabbit IgG isotype controls in stage EGK X blastoderms

Fig. S6: The specificity of labeling for the heterologous Bucky ball-antibody was investigated at three intrauterine stages comparative to the cell membrane marker pan-cadherin

Fig. S7: Megablast of chicken Bucky ball mRNA.

Fig. S8: Complete electrophoresis gel including the PCR products for the homeobox gene GAPDH and Bucky ball for the samples presented as cropped Figure 2b

Fig. S9: Protein structure prediction of chicken Bucky ball and germ plasm organizers from other species CC-BY-4.0 licence

Table S1: Scheme of Bucky ball synteny among vertebrate species (from Bontems[13])

Table S2: Experimental samples for immunohistochemical analyses

Table S3: Phosphate buffered salt solution

Table S4: List of intron-spanning primer sequences for RT-PCR

- LOCUS XM_040690219 3208 bp mRNA linear VRT
- 10-APR-2021 DEFINITION PREDICTED: Gallus gallus uncharacterized LOC420748,
- transcript variant X1, mRNA

- 1 gctgggcgca gcggctcagc gcaggcggga gcgccgcgtc gtgaacggtc gacggggagg 61 agccgcgtcc ccctcgccgt ccccgagcgc cgcggccagg tgggactgcc cctcgagcaa
- 121 acggccgggc cgtcagcccc gccccgggag aagcagcagg ggcgaggagc gacgtgtgta 181 ccaagagaaa aaaacattcc tgacagggaa tgtgttggaa agcagggagc gtctgtgtgt
- 241 ttatttccgt ttgggtccgt tagagcttca cagcctctac gcaccgaaag gaaggctggg 301 aatttccctt taagggtgaa agctttttgt ggtgaatgca ggaggtggga attccgagca
- 361 tgccagacgc acagtgagac acgcaggcgg ccaacggtca acacatgaga gcctctgtca 421 gaagaacgac acggtgtgcc tggataaagg cttttactgc tattcctatc ctcatatttt
- 481 gattctgccc cccattggat ggaagaggcc ccacatcacg gtcaaatgag ctgcaggtgg 541 aagagggagt aggcaagcag cagctgcctc ccataaagaa gtgtttaaaa aaaaaaatga
- 601 atactgcatc tgcttcagag agtggatcat attccacgaa ccactcaaga cccttcttct 661 atgcgcagcc aacagcgcaa caaccttttc aacatccatg gtacatcagc catacataca
- 721 atccatactg tgtgcctgca ccaggttttc gaggtggaca tccgtatttt ccgctttact 781 ctgttgcact ccatgagtac cctggatttt ttgttccaca gcatccagtg catgccagaa
- 841 ttaacagaag gccttatttt aatgctcccc caccctctcc catgttttat catgcaacgc 901 gatttagaca ctacagttct cctgggagaa aaacagagac aaaagaaaca cagactgatc
- 961 ctagacagcc tgaaaacaag caaaaaaagc atcaagatac ccatacagaa acgaaaggtt 1021 gtgatgcagg aaatatggcc tgtgtttctt ctggtatagg taaagagact gaaagtactt
- 1081 cagagaaaca agatctatct ggatcttcca ttgtggtaga cagggagttt cataatagtc 1141 cttccagctc tacacagtat agaaatcttc ctactggaag ctatgccttt gagaaggaag
- 1201 aggtgagaat agaatacgga aatggctctc cagctattca actgtggaag tcctttaaag 1261 aaactattcc tttgtatgat gtggcaagtg gtaaaccagt cccagagaac atggtgcagc
- 1321 gtgacgtatt ttctgttagc tcatgtgagg gaatgatata tggccctcat gaaggggaga 1381 aattggtgcc aggaacttac atagatgaga gaaaagctgt cctctcttca aaacagagtg
- 1441 ttgaaactat gcaagaaaaa gatgtccaag ataatgaagt gaagctggat gcagaaaagc 1501 aggtgactac aagtcaacgg gcaaaatcac ctccaggtga agccatggca gtgcaattag
- 1561 cagaactggc aagatctgtt accatagacc aaccagcagt aagacaggat gtgctagtag 1621 ctaagaaatc tagctctaaa agatccacag gttcaaaagc ttctcaagaa gagtccagct
- 1681 ttattcaaca agcaggattg cttccatcta gtatggaggt aatgagtgac ttgagttttc 1741 agcagaaaaa gctgaattta agccatagcg caaacaatga aagtcaaaca gatagaagca
- 1801 tttggtgtga agaatcagtt gagaagtatg ttccctctag cagctggctg gcttgtttgg 1861 ataatatgga tgctaactat agctatgaca tgtgtttgcc acaaaggaaa cgtcaaagcg
- 1921 tacttagtct ttcttctgat gacatgtcct ccagagagga tggctcatcc attgataatg 1981 ccccagtgtc ttattttgta cctgactatg tgcttcagaa aagcacgtat actttccaga
- 2041 aaagtacgga gggcttggag aaagagaaaa ttaaaagtgg tgggtccctc aatgaagatg 2101 cagtggtagg aagggagcag gtgaacagtt tggatggcca agatgtcaaa aattcttcaa
- 2161 ccatgaagat taaagaggct tccagtaaag gtagaaggct gagagtcctt cctagatctt 2221 ctagtcggaa aaagatctgt tctctcaaga aaaaagctgc caagagtttg tcagaaattg
- 2281 aggactctga agaatactct atgcaggtag aagaggagga tgaagatgaa gaggatgagg 2341 aggatgaaga tgatgatgtg gatgaaatag agtatttctt tcaagaagct gctccatatg
- 2401 ggatcttgat gccaagtaaa ggaaatattt acggacgagt tggccacagg atgctttgga 2461 agccaccaaa aaatgctttt ccaactcact taattagctg gcctgttcaa gagaagataa
- 2521 aaactaggag taggtttggt gaaaacattg gtgttgttta caaaccaaag gagaaagaac 2581 aagatgaagt tgtatatagt gactatgggt attatggaag aaagaggcca atggcaagaa
- 2641 gagaagaagg tgaacacaag cgaacactac agaaattctt gggaggaaga ctgttgaggg 2701 agagcatggg gataccacct gaagagtatt ggattagaag tggtgctaaa cccaaattca
- 2761 ctggacaaat acatggtagt ctctcacctc aagccaagag caaagaacaa gggtgcctgc 2821 ctccggttaa accaaagaag agaataggca agccccccact aaaacgcaga gacacaagat
- 2881 gtgaggtgga agaagtggaa gtgtgggagg tgcctaaaag gagtgtacgc aaaggaaaac 2941 gtggagcaag gaagtctctt tataagagaa gataactgca aatactgggg caggagggga
- 3001 gacaaatatc attgcatgaa accaaatgta aattattatt cttgtgtatt ttgtaacttg 3061 ctgtgcactc ttcaagggta agaagtgccc tccatctttg tggtgctgca agtaaactgt
- 3121 agaaagtatt taacactaaa ctaagtcact ttttgtaact aatgcatcca caatttaaat 3181 aataaactta atagaagtgc atttatga

Supplementary Figure S1: transcript XM 040690219 of LOC 420748

chicken Bucky ball

Predicted NLSs in query sequence

MNTASASESGSYSTNHSRPFFYAQPTAQQPFQHPWYISHTYNPYCVPAPG	50
FRGGHPYFPLYSVALHEYPGFFVPQHPVHARINRRPYFNAPPPSPMFYHA	100
TRFRHYSSPGRKTETKETQTDPRQPENKQKKHQDTHTETKGCDAGNMACV	150
SSGIGKETESTSEKQDLSGSSIVVDREFHNSPSSSTQYRNLPTGSYAFEK	200
EEVRIEYGNGSPAIQLWKSFKETIPLYDVASGKPVPENMVQRDVFSVSSC	250
EGMIYGPHEGEKLVPGTYIDERKAVLSSKQSVETMQEKDVQDNEVKLDAE	300
KQVTTSQRAKSPPGEAMAVQLAELARSVTIDQPAVRQDVLVAKKSSSKRS	350
TGSKASQEESSFIQQAGLLPSSMEVMSDLSFQQKKLNLSHSANNESQTDR	400
SIWCEESVEKYVPSSSWLACLDNMDANYSYDMCLPQRKRQSVLSLSSDDM	450
SSREDGSSIDNAPVSYFVPDYVLQKSTYTFQKSTEGLEKEKIKSGGSLNE	500
DAVVGREQVNSLDGQDVKNPSTMKIKEASSKGRRLRVLPRSSSRKKICSL	550
KKKAAKSLSEIEDSEEYSMQVEEEDEDEEDEEDEDDDVDEIEYFFQEAAP	600
YGILMPSKGNIYGRVGHRMLWKPPKNAFPTHLISWPVQEKIKTRSRFGEN	650
IGVVYKPKEKEQDEVVYSDYGYYGRKRPMARREEGEHKRTLQKFLGGRLL	700
RESMGIPPEEYWIRSGAKPKFTGQIHGSLSPQAKSKEQGCLPPVKPKKRI	750
GKPPLKRRDTRCEVEEVEVWEVPKRSVRKGKRGARKSLYKRR	792

Predicted monopartite NLS

Pos.	Sequence	Score
753	PPLKRRDTRC	8
778	RKGKRGARKSL	5.5

Predicted bipartite NLS

Pos.	Sequence	Score
675	RKRPMARREEGEHKRTLQK	6
748	KRIGKPPLKRRDTRCEVEEVEVWEVPKRSVRK	5.6
749	RIGKPPLKRRDTRCEVEEVEVWEVPKRSVRK	7.8
749	RIGKPPLKRRDTRCEVEEVEVWEVPKRSVR	5.5
749	RIGKPPLKRRDTRCEVEEVEVWEVPKRSVRKGK	5.7
749	RIGKPPLKRRDTRCEVEEVEVWEVPKRSVRKG	5.4
749	RIGKPPLKRRDTRCEVEEVEVWEVPKRSVRKGKR	6.7

zebrafish Bucky ball

Predicted NLSs in query sequence

MEGINNNSQPMGVGQPHHPVNHTRPFFYVQPPSQPYFMYQWPMNPYGHYG	50
FPGPALHFGRPYMAPYQFMQYPGYVIPHAPMQPIDYRRINPHYPSVASYD	100
LRVRHHFQNAGMHRETACSEVQTDPSDSVNKLIDKIESLKACELGSDKGP	150
NNVVSSTPDVVQGEKLTRLNEDSNLEVATKECKEDPVTRPTTYSDSAYDA	200
ESSQGRLDECVFSDVLPLDSSSVHEEEEEEEKDVNEEDEPQTVADEICSQ	250
NEMSASTTSNVFCSGVQSIADPTECHDLEKLGDEQKQDIPSADAAAVIEP	300
LISLSEDFDLPYQILRLPCNKTTTGLSLEREIDPLVYFDSPSTLLPPQNY	350
LSSIGSAYSYSYYPQVTQERQSVLSPSIDELSSRDEMFSTDVEDLEVVPG	400
HVYVGGGRLAEASDMPVRSRKELPSVDKTCSVCQKTCACCGSTLQDEVGM	450
CKMAEHSHPERDEMSDQDCDYDLEAEVRSNCESPRVSKRKCCSRHALPSC	500
GHHCAKHRHRKLLCEGQESCDLREQARVHPKGECCEEYGALAKADKRIQK	550
GALCRPCIEQQWREGVVSDQENWASCGAKPRSWRQVTGPQDQGRICVCLC	600
VKYAFVLFFFFFLPKGRTPLRRSTCKSIHQQRPRSEYNDYDETEFTYCQR	650
GRGMLSSLKC	660

Predicted monopartite NLS

Pos.	Sequence	Score
484	PRVSKRKCCS	9
484	PRVSKRKCCSR	10

Predicted bipartite NLS

Pos.	Sequence	Score
485	RVSKRKCCSRHALPSCGHHCAKHRHRK	6.9
485	RVSKRKCCSRHALPSCGHHCAKHRHR	5
485	RVSKRKCCSRHALPSCGHHCAKHRHRKLL	6
485	RVSKRKCCSRHALPSCGHHCAKHRHRKLLCE	5.4

Data generated by cNLS Mapper

Supplementary Figure S2: Amino acid sequences of the predicted chicken (left panel, chicken protein_id=XP_ 040518588.1) and zebrafish Bucky ball genes (right panel)

Highlighted positions indicate the predicted nuclear localization signals (NLS) and are detailed in boxes. The NLS [33] were predicted by cNLS mapper (https://nls-mapper.iab.keio.ac.jp/cgi-bin/NLS_Mapper_form.cg).

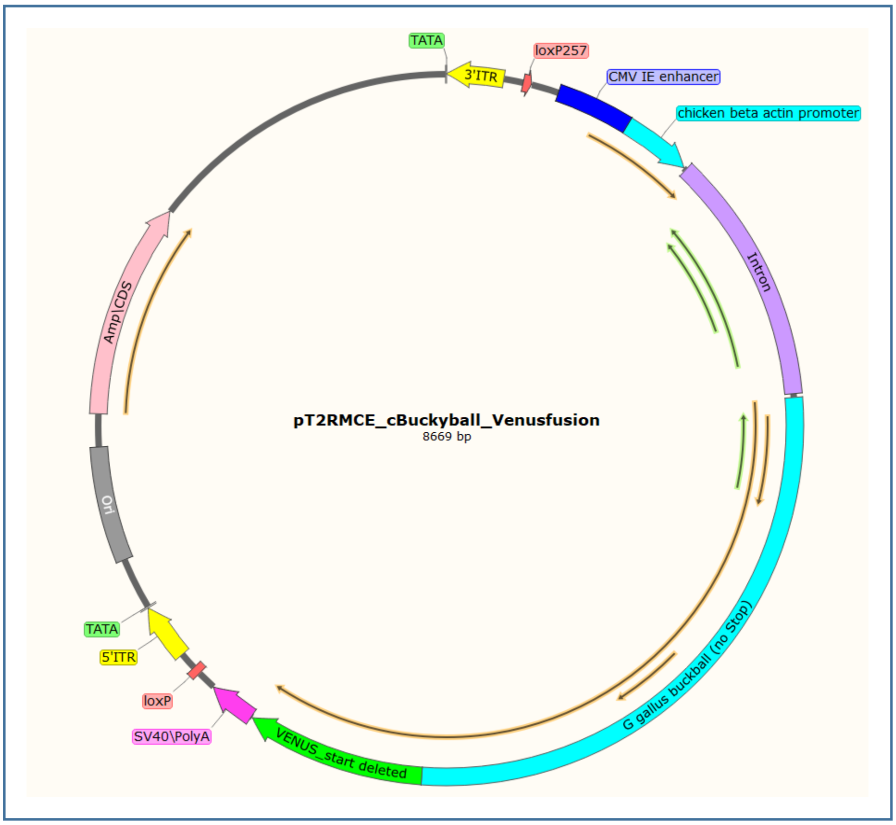

Supplementary Figure S3: Plasmid map of the chicken Loc 420748 cDNA without stop codon fused to Venus

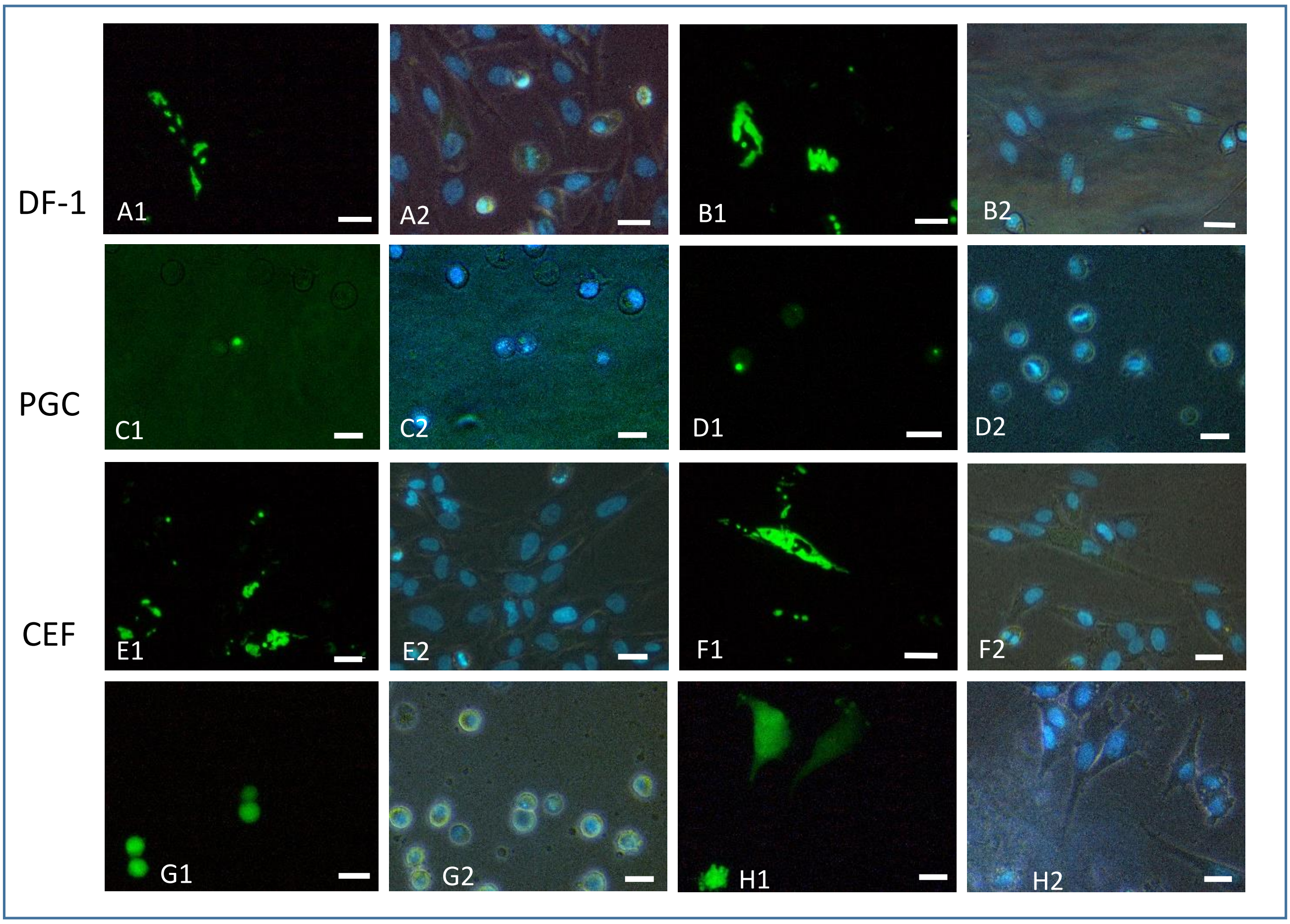

Supplementary Fig. S4: Expression of fusion constructs of Bucky ball and green fluorescent protein reporters in different chicken cell lines.

Aggregation of zebrafish Bucky ball (A1, C1, E1) and chicken-Bucky ball fusion proteins (B1, D1, F1) in DF1-cells (A, B), primordial germ cells (C, D), and chicken embryonic fibroblasts (E, F). In contrast, the reporter without a Bucky ball sequence shows a homogenous distribution in cytoplasm of PGCs (Fig. 2a G) and fibroblasts (Fig. 2a H). The Figs. 2a A2, B2, C2, D2, G2 are the corresponding phase contrast images. In Fig. E2, F2 and H2 the overlay of the phase contrast and the fluorescence is provided. In PGCs a nuclear localization of Bucky ball protein is evident, whereas in somatic DF1 cells and CEFs Bucky ball protein aggregates appear in nuclei and cytoplasm. In Figure C2 and D2 Hoechst 33342 is added as nuclear marker stain. (Scales: 20 µm).

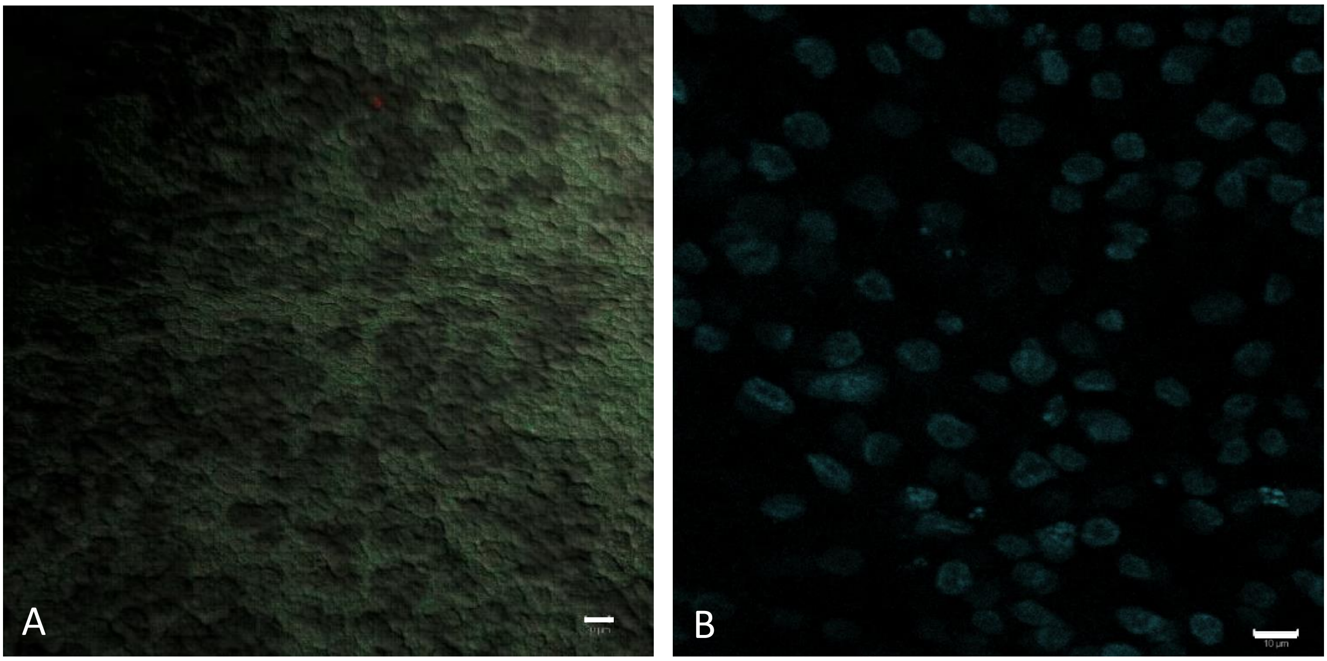

Supplementary Figure: S5: Specificity controls without primary antibody and anti-rabbit IgG isotype controls in stage EGK X blastoderms

A: Immunofluorescnce control samples with FITC-anti-rb and A555-anti-gp secondary antibodies without primary antibodies for Bucky ball and CVH in each individual run of immunolabeling did not show any label above background, example shown from a stage EGK X blastoderm, scale 20 µm

B: The isotype anti-rabbit IgG control did not show any labeling at the optimized aquisition adjustments for Bucky ball detection adding to the nuclear counterstaining, scale 10 µm.

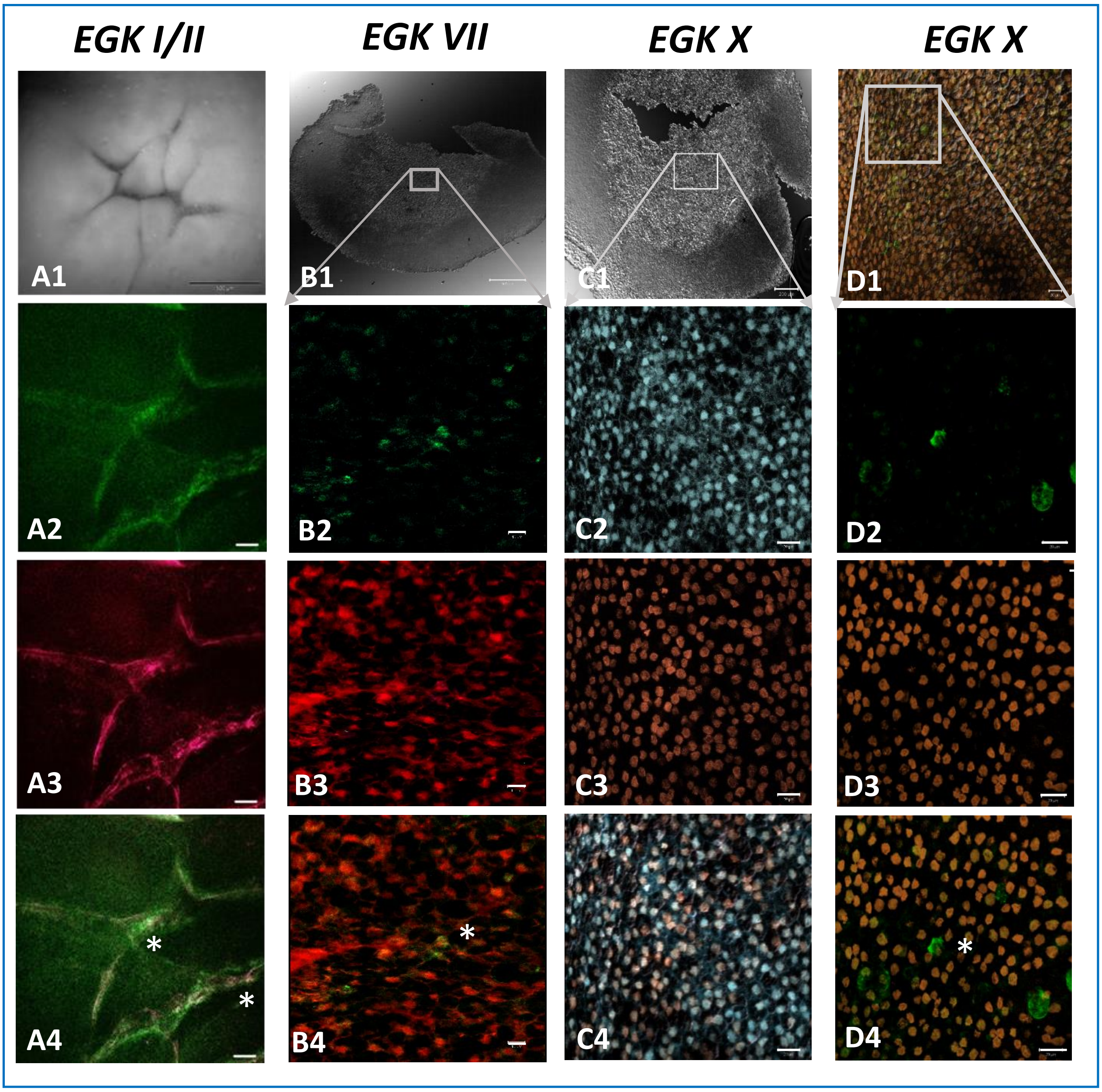

Supplementary Figure S6: The specificity of labeling for the heterologous Bucky ball-antibody was investigated at three intrauterine stages comparative to the cell membrane marker pan-cadherin

Pan-cadherin labeling at intrauterine stages EGK I (A3, A4, pink label), EGK VII (panel B3, B4 red label) and EGK X (panel C2, C4, light blue label) with partly co-labeling for Bucky ball (A4, B2, and D2, green label). Nuclear marker SIR is shown in C3, and D3, orange label).
In EGK I/II Bucky ball is aggregating in the cytoplasm and accumulated into furrow cleavages. The cell membrane marker pan-cadherin labels the furrows, but there is only partional co-localization to Bucky ball (panel A4 *). Colocalization to Bucky ball appears in few cells in defined cells of later stages at EGK VII and EGK X (B4, D4). In EGK X, Bucky ball is clearly only cytoplasmatically labeled and pan-cadherin is found at cell membranes with additional nuclear labeling. The distribution of Bucky ball in scattered central cells in stage EGK X epiblast, corresponds to the localization of PGCs in the center of the epiblast is well documented by CVH, DAZL, and SSEA1 labeling. Around 50 cells are expected as PGCs with specific labeling of all three antibodies at that stage [18, 26, 43].
Scale in A1 = 500 µm, B1 and C1= 2000 µm, D1= 50 µm, A2-D4 =20 µm;

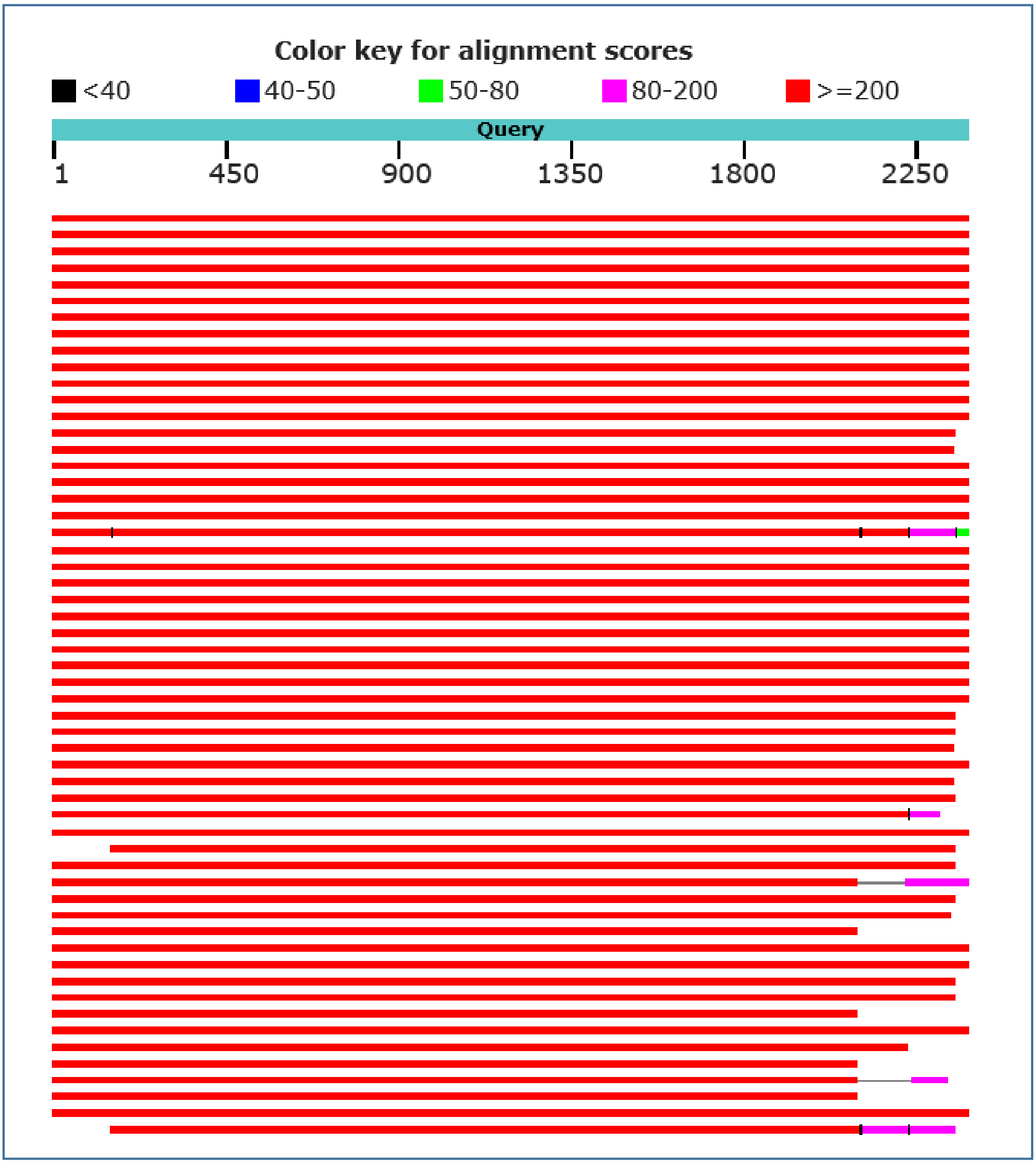

Supplementary Figure S7: Megablast of chicken Bucky ball mRNA

Blast (Blastn) analysis revealed a very high conservation of the Bucky ball gene within archelosauria. A total of 225 matching sequences were found, repre-senting 109 species with 95 birds, as well as 14 crocodiles and turtles.

S7a: Distribution of the top Blast Hits is shown.

Supplementary Figure S7b: Blast data of Bucky ball homology of 109 archelosauria

NIH U.S. National Library of Medicine
National Center for Biotechnology Information

Log in

COVID-19 Information

Public health information (CDC) | Research information (NIH) | SARS-CoV-2 data (NCBI) | Prevention and treatment information (HHS) | Español

BLAST® » blastn suite » results for RID-STTUP1FG016

Home Recent Results Saved Strategies Help

Edit Search Save Search Search Summary

How to read this report? BLAST Help Videos Back to Traditional Results Page

Job Title	Nucleotide Sequence
RID	STTUP1FG016 Search expires on 11-12 16:50 pm Download All
Program	BLASTN Citation
Database	nt See details
Query ID	lcl|Query_42101
Description	None
Molecule type	dna
Query Length	2379
Other reports	Distance tree of results MSA viewer

Filter Results

Organism only top 20 will appear exclude

Type common name, binomial, taxid or group name

+ Add organism

Percent Identity to E value to Query Coverage to

Filter Reset

Descriptions Graphic Summary Alignments Taxonomy

Sequences producing significant alignments

Download Select columns Show 500

select all 1 sequences selected

GenBank Graphics Distance tree of results MSA Viewer

Feedback

Description	Scientific Name	Max Score	Total Score	Query Cover	E value	Per. Ident	Acc. Len	Accession
PREDICTED: Gallus gallus uncharacterized LOC420748 (LOC420748), transcript variant X3, mRNA	Gallus g...	4377	4377	100%	0.0	99.87%	3725	XM_040662656.1
PREDICTED: Gallus gallus uncharacterized LOC420748 (LOC420748), transcript variant X2, mRNA	Gallus g...	4377	4377	100%	0.0	99.87%	6118	XM_040662655.1
PREDICTED: Gallus gallus uncharacterized LOC420748 (LOC420748), transcript variant X1, mRNA	Gallus g...	4377	4377	100%	0.0	99.87%	3209	XM_040662654.1
PREDICTED: Gallus gallus uncharacterized LOC420748 (LOC420748), transcript variant X3, mRNA	Gallus g...	4377	4377	100%	0.0	99.87%	3725	XM_040690232.1
PREDICTED: Gallus gallus uncharacterized LOC420748 (LOC420748), transcript variant X2, mRNA	Gallus g...	4377	4377	100%	0.0	99.87%	6117	XM_040690227.1
PREDICTED: Gallus gallus uncharacterized LOC420748 (LOC420748), transcript variant X1, mRNA	Gallus g...	4377	4377	100%	0.0	99.87%	3208	XM_040690219.1
PREDICTED: Phasianus colchicus uncharacterized LOC116238596 (LOC116238596), transcript variant X2, mRNA	Phasian...	3980	3980	100%	0.0	96.89%	2918	XM_031608366.1
PREDICTED: Phasianus colchicus uncharacterized LOC116238596 (LOC116238596), transcript variant X1, mRNA	Phasian...	3980	3980	100%	0.0	96.89%	4674	XM_031608365.1
PREDICTED: Meleagris gallopavo uncharacterized LOC104911574 (LOC104911574), transcript variant X2, mRNA	Meleagr...	3925	3925	100%	0.0	96.47%	3111	XM_019616674.1
PREDICTED: Meleagris gallopavo uncharacterized LOC104911574 (LOC104911574), transcript variant X1, mRNA	Meleagr...	3925	3925	100%	0.0	96.47%	2925	XM_010713049.2
PREDICTED: Centrocercus urophasianus uncharacterized LOC122159443 (LOC122159443), transcript variant ...	Centroc...	3895	3895	100%	0.0	96.26%	2651	XM_042817028.1
PREDICTED: Lagopus leucura uncharacterized LOC122189505 (LOC122189505), transcript variant X2, mRNA	Lagopu...	3884	3884	100%	0.0	96.17%	2715	XM_042888744.1
PREDICTED: Lagopus leucura uncharacterized LOC122189505 (LOC122189505), transcript variant X1, mRNA	Lagopu...	3884	3884	100%	0.0	96.17%	2907	XM_042888743.1
PREDICTED: Meleagris gallopavo uncharacterized LOC104911574 (LOC104911574), transcript variant X3, mRNA	Meleagr...	3877	3877	98%	0.0	96.58%	2632	XM_019616675.1
PREDICTED: Centrocercus urophasianus uncharacterized LOC122159443 (LOC122159443), transcript variant ...	Centroc...	3849	3849	98%	0.0	96.45%	2493	XM_042817029.1
PREDICTED: Numida meleagris uncharacterized LOC110393692 (LOC110393692), transcript variant X2, mRNA	Numida ...	3757	3757	100%	0.0	95.18%	7786	XM_021386846.1
PREDICTED: Numida meleagris uncharacterized LOC110393692 (LOC110393692), transcript variant X1, mRNA	Numida ...	3757	3757	100%	0.0	95.18%	7979	XM_021386845.1
PREDICTED: Coturnix japonica uncharacterized LOC107309880 (LOC107309880), transcript variant X2, mRNA	Coturnix...	3557	3557	100%	0.0	93.75%	2715	XM_032442566.1
PREDICTED: Coturnix japonica uncharacterized LOC107309880 (LOC107309880), transcript variant X1, mRNA	Coturnix...	3557	3557	100%	0.0	93.75%	2818	XM_015855039.2
Meleagris gallopavo genome assembly, chromosome: 6	Meleagr...	3243	3968	99%	0.0	96.72%	51422372	HG999686.1
PREDICTED: Cygnus olor uncharacterized LOC121066658 (LOC121066658), transcript variant X3, mRNA	Cygnus ...	3214	3214	100%	0.0	91.15%	8371	XM_040550371.1
PREDICTED: Cygnus atratus uncharacterized LOC118250856 (LOC118250856), transcript variant X2, mRNA	Cygnus ...	3208	3208	100%	0.0	91.10%	2836	XM_035552360.1
PREDICTED: Cygnus atratus uncharacterized LOC118250856 (LOC118250856), transcript variant X1, mRNA	Cygnus ...	3208	3208	100%	0.0	91.10%	2907	XM_035552358.1
PREDICTED: Anser cygnoides domesticus uncharacterized LOC106045510 (LOC106045510), mRNA	Anser c...	3199	3199	100%	0.0	91.02%	7177	XM_013196072.1
PREDICTED: Anas platyrhynchos uncharacterized LOC101800106 (LOC101800106), transcript variant X3, mRNA	Anas pl...	3083	3083	100%	0.0	90.13%	4580	XM_038175493.1
PREDICTED: Anas platyrhynchos uncharacterized LOC101800106 (LOC101800106), transcript variant X2, mRNA	Anas pl...	3083	3083	100%	0.0	90.13%	3764	XM_038175492.1
PREDICTED: Anas platyrhynchos uncharacterized LOC101800106 (LOC101800106), transcript variant X1, mRNA	Anas pl...	3083	3083	100%	0.0	90.13%	4428	XM_038175491.1
PREDICTED: Aptenodytes forsteri uncharacterized LOC103894172 (LOC103894172), mRNA	Aptenod...	3022	3022	100%	0.0	89.65%	2376	XM_019470530.1
PREDICTED: Gavia stellata uncharacterized LOC104252952 (LOC104252952), mRNA	Gavia st...	2964	2964	100%	0.0	89.18%	2385	XM_009808255.1
PREDICTED: Apteryx rowi uncharacterized LOC112967444 (LOC112967444), transcript variant X2, mRNA	Apteryx ...	2931	2931	100%	0.0	88.94%	3178	XM_026068594.1
PREDICTED: Pelecanus crispus uncharacterized LOC104026434 (LOC104026434), mRNA	Pelecan...	2926	2926	98%	0.0	89.31%	2373	XM_009482791.1
PREDICTED: Struthio camelus australis uncharacterized LOC104147248 (LOC104147248), mRNA	Struthio ...	2852	2852	98%	0.0	88.65%	2355	XM_009679790.1
PREDICTED: Phaethon lepturus uncharacterized LOC104616043 (LOC104616043), mRNA	Phaetho...	2846	2846	98%	0.0	88.71%	2454	XM_010295494.1
PREDICTED: Dromaius novaehollandiae uncharacterized LOC112983565 (LOC112983565), mRNA	Dromai...	2835	2835	100%	0.0	88.21%	2700	XM_026100758.1
PREDICTED: Balearica regulorum gibbericeps uncharacterized LOC104631619 (LOC104631619), mRNA	Balearic...	2824	2824	98%	0.0	88.50%	2562	XM_010303851.1
PREDICTED: Cariama cristata uncharacterized LOC104156814 (LOC104156814), mRNA	Cariam...	2802	2802	98%	0.0	88.31%	2514	XM_009709772.1
PREDICTED: Apteryx rowi uncharacterized LOC112967444 (LOC112967444), transcript variant X1, mRNA	Apteryx ...	2780	2879	96%	0.0	89.33%	4770	XM_026068593.1
PREDICTED: Haliaeetus albicilla uncharacterized LOC104323560 (LOC104323560), mRNA	Haliaeet...	2776	2776	100%	0.0	87.85%	2367	XM_009927271.1
PREDICTED: Apteryx australis mantelli uncharacterized LOC106489003 (LOC106489003), mRNA	Apteryx ...	2760	2760	92%	0.0	89.41%	2366	XM_013948118.1
PREDICTED: Aquila chrysaetos chrysaetos uncharacterized LOC115339560 (LOC115339560), mRNA	Aquila c...	2715	2715	98%	0.0	87.78%	2995	XM_030009722.2
PREDICTED: Pygoscelis adeliae uncharacterized LOC103923978 (LOC103923978), mRNA	Pygosc...	2697	2866	94%	0.0	89.98%	2253	XM_009332414.1
PREDICTED: Chlamydotis macqueenii uncharacterized LOC104486194 (LOC104486194), mRNA	Chlamy...	2676	2676	98%	0.0	87.38%	2457	XM_010128803.1
PREDICTED: Phalacrocorax carbo uncharacterized LOC104040700 (LOC104040700), partial mRNA	Phalacr...	2675	2675	97%	0.0	87.42%	2350	XM_009503751.1
PREDICTED: Nipponia nippon uncharacterized LOC104012137 (LOC104012137), mRNA	Nipponi...	2665	2665	87%	0.0	89.73%	2250	XM_009464491.1
PREDICTED: Columba livia uncharacterized LOC110357017 (LOC110357017), mRNA	Columb...	2643	2643	100%	0.0	86.96%	2790	XM_021284129.1
PREDICTED: Opisthocomus hoazin uncharacterized LOC104328673 (LOC104328673), mRNA	Opistho...	2639	2639	100%	0.0	86.82%	2364	XM_009933704.1
PREDICTED: Tyto alba uncharacterized LOC104356678 (LOC104356678), mRNA	Tyto alba	2608	2608	98%	0.0	86.98%	2624	XM_009970741.3
PREDICTED: Tauraco erythrolophus uncharacterized LOC104374577 (LOC104374577), mRNA	Tauraco...	2569	2569	98%	0.0	86.57%	2358	XM_009980715.1
PREDICTED: Charadrius vociferus uncharacterized LOC104287496 (LOC104287496), mRNA	Charadr...	2555	2555	87%	0.0	88.88%	2238	XM_009886212.1

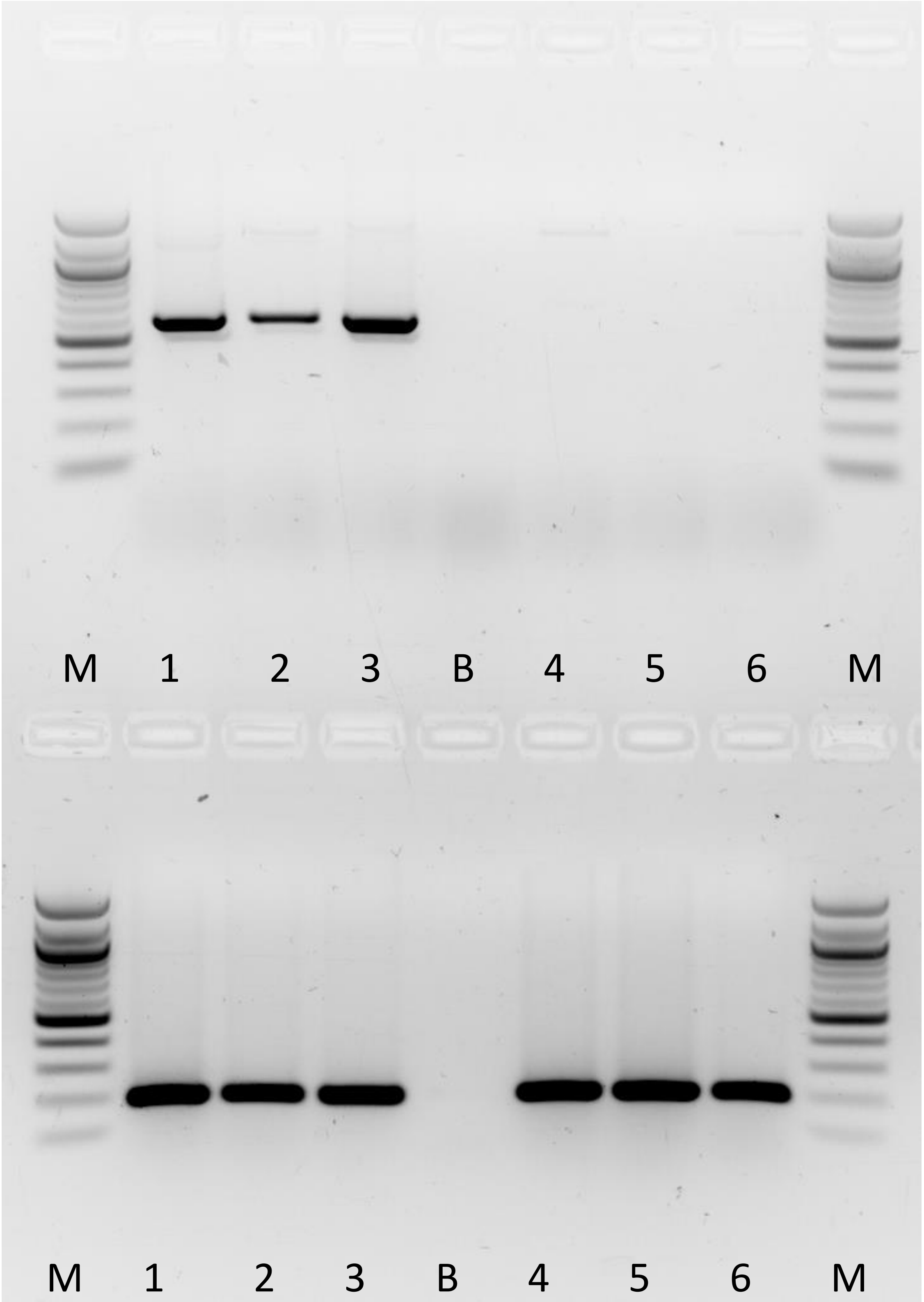

Supplementary Figure S8: Complete gel electrophoresis including the PCR products for Bucky ball (upper panel) and the homebox gene GAPDH (lower panel) for the samples presented as condensed form in Figure 2b

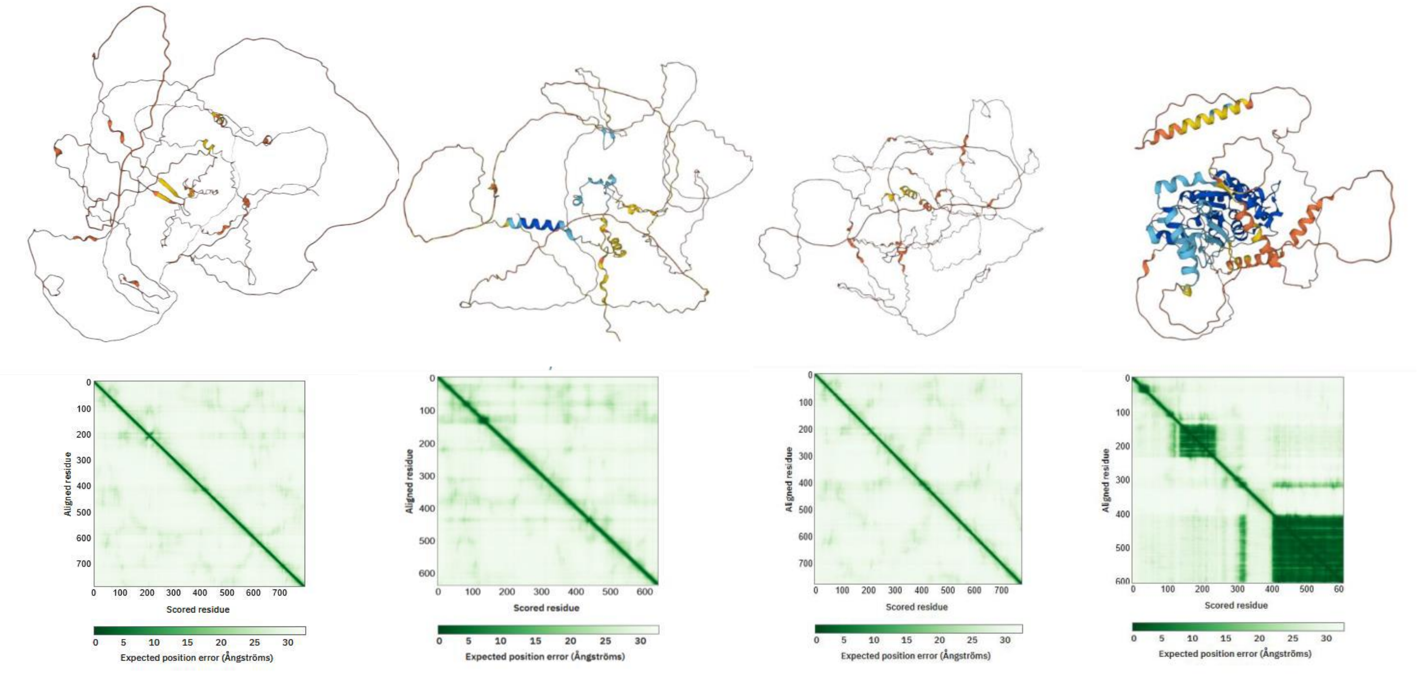

Fig. S9: Protein structure prediction of chicken Bucky ball and germ plasm organizers from other species

AlfaFold (https://alphafold.ebi.ac.uk/; assessed on 23.08.2020) predicted the protein structure (top) and calculated an expected position error (bottom) for the indicated proteins. The per-residue confidence scoring strongly suggested that chicken Bucky ball, zebrafish Bucky ball, and Xvelo1 are basically unstructured in isolation, as characteristic for IDPs. Oskar is predicted to contain unstructured as well as alpha helical domains. Predicted aligned error: The color at position (x, y) indicates AlphaFold’s expected position error at residue x, when the predicted and true structures are aligned on residue y.

https://creativecommons.org/licenses/by/4.0/

Table S1. Scheme of *Bucky Ball* Synteny among Vertebrate Species (from Bontems [18])

**DANIO chr 2**	Cadher. 6	port kinase 2	Riken MESR6	retro virus	melanocort recpt 4 zMC4 E.15515	glutamate deshydro LOC558710 XR_029813.1	ring finger 152 zgc:110537	buc LOC560382						ring finger 31 E.63673	XK related prot 4 id:ibd5007 E.19080
**TETRA uk**							ring finger 152 G.16536001	buc G.16538001	Buc-like G.16539001	Kelch rep G.16540001	KIAA0241 G.16541001	U6 sn RNA G.16542001	proteosome PSME2 G.16543001	ring finger 31 G.16545001	
**FUGU sca 96**							ring finger 152 S.121584	buc S.160955	Buc-like S.159880	Kelch rep S.121597	KIAA0241 S.121600		proteosome PSME2 S.121605	ring finger 31 S.121570	
**XENOP sca 254**		PTHB1 BBS9 E. 21125			NT5C3 E.21122		retin pigm 9 LOC496684	Xvelo1 E.21119		Kelch rep E.21117	KIAA0241 E.21115				
**GALLUS chr 2**		PTHB1 LOC 420745			NT5C3 5NT3_CHICK E.12192	FK506 NP_990178.1 E.12193	retin pigm 9 LOC420747	buc LOC420748		Kelch rep KBTBD2 E.12202	KIAA0241 LOC420750	U6 sn RNA LSM5 E.12209			
**COW chr 4**		BBS9 PTHB1 LOC 535387	retin pigm 9 LOC7 83842	buc-like LOC783842	NT5C3 NP_ 1032686.1	FK506 FKBP9_ BOVIN E.16707	retin pigm 9 GSC.42811	buc GSC.42786		Kelch rep LOC514920	KIAA0241 LOC534141				
**CANIS chr 14**		PTHB1 482393 E.3161	retin pigm 9 E. 24731	buc-like 58820	NT5C3 475277 E.3154	FK506 475276 E.3151	retin pigm 9 E.3158	buc GSC.91809		Kelch rep 482391 E.3147	KIAA0241 482390 E.3143				
**HUMAN chr 7**					NT5C3 E.122643	FK506 FKBP9 CCDS5439 E.122642	retin pigm 9 Q9BZU6_ HUMAN	buc EST86245		Kelch rep KBTBD2	KIAA0241 HGNC E.105778	U6 sn RNA LSM5 YER146W E.106355			

Table S2: Experimental samples for immunohistochemical analyses

	No of oocytes and embryos with recovered stages (according to Eyal-Giladi & Kochav [1])							Eggs incubated for 12h, stages according to Ham-burger & Hamilton [46]	No of controls without primary antibody + anti-rb FITC	No of Isotype and pan-cadherin controls	No of Controls - prim. ab + anti-rb FITC /anti-mc Cy5
	**Matured oocytes and zygotes**	**EK I / EKII**	**EK III**	**EKIV**	**EK V/ VI**	**EK VII/VIII**	**EK X**	**HH 5-8**			
No Experiments including respective samples	4	5	3	4	3	3	7	5	14	5	7
Totally analyzed samples	25	21	10	7	10	16	55	45	15	6	7
Damaged and not evaluated samples	13	5	1	0	0	1	6	7	0	0	0
No of unequivocally evaluated samples	12	15	9	7	10	15	49	38	15	6	7

Table S3: Phosphate buffered salt solution

	Stock	Fixation	Immunostaining
Disodiumhydrogenphosphate	0.2 M	0.1 M	0.02 M
Sodiumdihydrogenphoshate	0.2 M	0.1 M	0.02 M
Sodiumchloride	0.77 M	0.385 M	0.077 M

Table S4: List of intron-spanning primer sequences for rt-PCR

Gen	Product size	Forward primer	Reverse primer
Bucky ball	594	5-ATCCATACTGTGTGCCTGCA-3	5- CCATGTTCTCTGGGACTGGT-3
GAPDH	200	5'- CCTCTCTGGCAAAGTCCAAG -'3	5'- CATCTGCCCATTTGATGTTG -'3
